# Comparative transcriptome profiling to unravel the key molecular signalling pathways and drought adaptive plasticity in shoot borne root system of sugarcane

**DOI:** 10.1038/s41598-023-39970-1

**Published:** 2023-08-08

**Authors:** R. Valarmathi, H. K. Mahadeva Swamy, C. Appunu, G. S. Suresha, K. Mohanraj, G. Hemaprabha, C. Mahadevaiah, V. Ulaganathan

**Affiliations:** Division of Crop Improvement, ICAR-Sugarcane Breeding Institute, Coimbatore, 641007 India

**Keywords:** Biotechnology, Molecular biology

## Abstract

Sugarcane root system comprises of superficial sett roots as well as deeply-penetrating shoot borne roots (SBR) with latter being the permanent root system. In sugarcane, the healthy SBR contributes to a better crop yield and it also helps to produce multiple ratoon crops after the harvest. There is a dearth of in-depth knowledge on SBR system architecture and its functional role in modern day commercial hybrids. A comprehensive phenotypic, anatomical and whole transcriptome profiling, conducted between the commercial sugarcane hybrids and a wild germplasm *Erianthus*, found a developmental delay in both initiation and establishment of the SBR in commercial hybrid compared to *Erianthus*. The SBR system in *Erianthus* proved to be an extensive drought-adaptive root system architecture that significantly contributes to drought tolerance. On the other hand, SBRs in the commercial hybrids showed an irreversible collapse and damage of the root cells under drought stress. The outcomes from the comparative analysis of the transcriptome data showed a significant upregulation of the genes that regulate important stress signalling pathways viz., sugar, calcium, hormone signalling and phenylpropanoid biosynthesis in the SBRs of *Erianthus*. It was found that through these key signalling pathways, *Erianthus* SBRs triggered the downstream signalling cascade to impart physiological responses like osmoprotection, modification of the cell walls, detoxification of reactive oxygen species, expression of drought responsive transcription factors, maintenance of cell stability and lateral root development. The current study forms a basis for further exploration of the Shoot Borne Root system as a valuable breeding target to develop drought tolerant sugarcane genotypes.

## Introduction

Globally, the frequency of abiotic stresses such as drought, salinity, waterlogging, high temperature and other extreme weather conditions is increasing every year^1^. Sugarcane is one of the most economically important commercial crops and is a major source of sugar across the globe. However, drought is emerging as a major problem that affects the productivity and production of sugarcane worldwide. India, with an area of 5.1 million hectares, is one of the leading countries in sugarcane cultivation. At present, about 0.29 million hectare landmass, under sugarcane cultivation in India, is reported to be drought-prone^2–6^. Drought stress, particularly at the formative phase of the crop, inhibits more than 50% of its yield^7,8^. The use of tolerant and better adaptive varieties will help in achieving sustainable productivity under drought stress condition^8^.

In order to develop drought tolerant or adaptive crops, those plants with better root system architecture have been targeted widely in breeding programs^9^. Roots are the first organs in plant body to sense the drought stress and initiate the stress signalling cascade. The signalling cascade causes metabolic and molecular changes in the plant so as to adapt itself under extreme environmental conditions^10,11^. Extensive studies have been conducted earlier to unravel the root molecular signalling pathways and its regulatory mechanisms so that the root strategies to maintain functions under limited water availability can be understood in a better manner^10–13^.

Sugarcane root system is highly divergent in nature as it forms different types of root systems during different phases of its development^14^. The primary root i.e., sett roots, is formed from the root eyes of the vegetative setts, during bud germination. It supports seedling development while it eventually degrades within 60–90 days of germination. After 5–7 days of sett planting, thicker and permanent Shoot Borne Roots (SBR) arise from the consecutive shoot nodes. This root system is unique to monocots and forms the permanent root system in sugarcane by contributing to adult fitness and final crop yield^14,15^.

In maize, SBR system has been extensively studied using the developmental mutants and is shown to exhibit lodging resistance, better crop yield and also improved water use efficiency^16–18^. Sugarcane, with a tall stature of 2–3 m and a huge biomass, requires an efficient root system so as to provide the much-required mechanical support in addition to its role on water and nutrient uptake^14,19^. Hence understanding sugarcane SBRs could serve as a valuable target for breeding drought tolerant varities^20,21^. In sugarcane, no reports have been published so far on the physiological and molecular mechanisms that underlie the functions of the SBR system.

In the study conducted by the same author earlier, a few commercial sugarcane genotypes were screened along with wild germplasm (*Erianthus arundinaceus*) for drought tolerance-related traits. The study showed better SBR system in wild germplasm with high tolerance towards extreme drought conditions^15^. The present study was conducted to understand the developmental, morphological, drought-adaptive anatomical plasticity and molecular insights about the transcriptomics of sugarcane SBRs. The morpho-anatomical basis of SBR development showed significant differences in both initiation and establishment of the SBRs between the commercial sugarcane genotypes and its wild relative (*E. arundinaceus*) at various developmental stages. Further, a comparative anatomy and whole transcriptome analysis of the SBRs, under drought stress conditions, revealed novel insights about the molecular signalling and key pathways that confer drought-adaptive plasticity by the SBRs.

## Materials and methods

### Phenotyping of SBR initiation and establishment in sugarcane

In the study, morphological phenotyping was carried out to determine the time course of initiation and establishment of SBRs. To phenotype the initial development of SBRs, two clones of sugarcane wild germplasm *Erianthus arundinaceus* (IND 04-1335 and SES 288) from the world germplasm collection, maintained at ICAR Sugarcane Breeding Institute, and a popular commercial sugarcane variety i.e., Co 86032 were used. Single-bud vegetative setts from each genotype (ten replications per genotype) were planted in pots (measuring 12 inches upper diameter, 10 inches height and 8 inches base diameter) containing red soil, sand and farmyard manure (1:1:1). The experiments were carried out at the controlled greenhouse facility of ICAR Sugarcane Breeding Institute, Coimbatore (77° E longitude and 11° N latitude, Elevation 427 m) (1500–1800 µmol m^−2^ s^−1^ light intensity, photoperiod of 16 h light and 8 h dark, temperature of 30 °C ± 2 °C with ~ 75% relative humidity). The settlings were phenotyped for emergence and the development of SBRs at different days such as 20, 30 and 40 days after planting (DAP). For root phenotyping, the settlings were laid down on top of root washing mesh with 4.50 mm aperture and were gently washed using a sprayer in order to remove all the soil particles^22^. For anatomical observation, the first shoot node was transversely dissected to observe the initiation of SBR primordia. Hand sections of the first node from ten replications were visualised and photographed using Carl Zeiss Axiolab 5 microscope (Zeiss, Germany), attached with Axiocam 208 color camera.

### Morphology and anatomical phenotyping of the SBRs under drought condition

To phenotype SBRs under drought condition, two *E. arundinaceus* clones (IND 04-1335 and SES 288) and two commercial sugarcane varieties such as Co 86032 and Co 775 (drought susceptible check) were taken. Single-budded setts from both the groups (of ten replications each) were planted in pots (measuring 18 inches upper diameter, 15 inches height and 10 inches lower diameter) containing soil, sand and farmyard manure (1:1:1)^23^. The greenhouse conditions described in the previous section were maintained. Before imposing drought stress, the soil moisture content at field capacity and permanent wilting point were determined using a pressure plate apparatus^24^. Drought was imposed upon 45-days’ old plants by withholding irrigation along with a set of controls that are irrigated to field capacity. Root phenotyping was conducted in two stages for morphological and anatomical variations in which the first stage was performed after 15 days of drought exposure and the second stage was performed after 30 days of drought exposure. The soil moisture content was measured based on gravimetric method at both the stages of sampling. Both length and the biomass of SBRs were measured manually while the anatomy of the SBRs was studied using hand section taken at 10–20 cm from the root base. The hand sections of roots were also visualised with phloroglucinol-HCl staining. Ultrastructures of the cellular changes in Co 86032 as well as IND 04-1335 were observed using the Scanning Electron Microscope (SEM) (FEIQuanta250, Icon analytical, FEI, USA). For SEM imaging the sections measuring 4–5 mm thickness were selected and gold coated with a sputter coater and then mounted on an aluminium stub using a double-side adhesive tape. Then the samples were scanned and the selected regions were photographed at 500×. The SPSS statistical package (SPSS Inc., Chicago, IL, USA) was used to compute the mean values and standard errors for the root traits. The significant difference between the groups were determined using one-way ANOVA with Duncan's multiple range test. The bar plots were generated using the ggplot function in ggplot2 package of R software, version 4.1.2.

### Transcriptome profiling of the SBRs exposed to drought condition

The SBRs of both Co 86032 and *E. arundinaceus* clone (IND 04-1335) were subjected to whole transcriptome sequencing. The roots were collected from both control and drought-stressed plants on 16th day of exposure to drought stress and frozen in liquid nitrogen. The frozen root samples were ground using liquid nitrogen and total RNA was then extracted using Trizol Reagent (Biobasic Inc., Canada) and purified using RNeasy Plant Mini Kit (Qiagen, Valencia, CA). After verifying the quality of total RNA followed by the removal of rRNA, mRNA was enriched using oligo (dT) beads. The enriched mRNA was then fragmented randomly using fragmentation buffer, followed by cDNA synthesis using random hexamers and reverse transcriptase. After the synthesis of first-strand, a custom second-strand synthesis buffer (Illumina) was added to the solution with dNTPs, RNase H and *Escherichia coli* polymerase I to generate the second strand by nick-translation. AMPure XP beads were used to purify the cDNA. The final cDNA library was prepared after purification, terminal repair, A tailing, ligation of the sequencing adapters, size selection and PCR enrichment. Library concentration was quantified using the Qubit 2.0 fluorometer (Life Technologies, USA) and then diluted to 1 ng/µl. The insert size was checked on Agilent 2100 Tapestation and quantified to a great accuracy by quantitative PCR (Q-PCR) (library activity > 2 nM). The library was then sequenced using the Illumina platform (HiSeq 2500) at Yazh Xenomics, Coimbatore, Tamil Nadu, India.

### Data quality check and data filtering

The Illumina reads were adapter-trimmed and quality-controlled using the BBduk tool, BBTools package v38.90 (BBMap-Bushnell B.-sourceforge.net/projects/bbmap/). Then, the reads were quality-trimmed to Phred score greater than 20 (Q20) and a minimum length of 50 bases. The mate pairs of high quality (i.e. both forward and reverse reads passed QC) were only used for conducting the downstream analysis. The clean reads were quality-assessed using FastQC tool v0.11.9 and raw reads were submitted to the NCBI-SRA database (PRJNA937025).

### De novo transcriptome assembly

High-quality reads of control and the stressed samples of both the genotypes i.e., IND 04-1335 and Co 86032 were then combined and assembled de novo into transcriptome using the assembler, Trinity v2.12.0^25^. The assembled transcripts were then clustered into a non-redundant set of transcripts using CD-HIT-EST^26^ at 90% identity and 95% query coverage. Completeness of the assembly were checked by evaluating the recovery of the most highly-conserved orthologs in the transcriptome using BUSCO (Benchmarking Universal Single-Copy Orthologs) v5.1.2^27^.

### Read quantification and differential gene expression analysis

RSEM (RNA-Seq by Expectation–Maximization) tool v1.3.3^28^ was applied to quantify the expression level of the transcripts. RSEM algorithm was used with expectation–maximization technique. The transcript abundance was estimated as transcripts per million mapped reads (TPM) and Fragments Per Kilobase of transcript per Million mapped reads (FPKM). The transcript-level differential expression analysis was conducted using DESEQ2 v1.30.1^29^ in R on the matrix of raw read counts. Differentially-expressed significant transcripts were then identified based on the cut-off value of FDR (False Discovery Rate) adjusted *p*-value < 0.01 and log_2_FC ≥ 1 (Upregulated genes) and log_2_FC ≤ − 1 (Downregulated genes).

### Functional annotation of the differentially expressed genes

The differentially-expressed transcripts were extracted and a comprehensive gene functional annotation was conducted. Functional annotation was carried out using Trinotate tool v3.2.1 (https://trinotate.github.io/)^30^. The following databases were used for annotation viz*.,* NCBI-nr, Pfam, KEGG, GO, KOG and MapMan. The annotations of the DEGs against NCBI-nr (https://www.ncbi.nlm.nih.gov/refseq/about/nonredundantproteins) was carried out based on the e-value threshold of 1e-5. Pfam (http://pfam.xfam.org/) database was used with HMMer program with an e-value threshold of 0.01. From the GO (Gene Ontology) annotationsGO terms were obtained using Blast2GO v2.5^31^ at an e-value threshold of 1e-6. The GO annotations were then classified and visualized using the web tool WEGO (Web Gene Ontology Annotation Plot) (https://wego.genomics.cn/)^32^. For KOG (EuKaryotic Orthologous Groups) annotations, the transcripts were blasted against the KOG database with an e-value threshold of 1e-3 and classified using web MGA server^33^. KEGG (Kyoto Encyclopedia of Genes and Genomes) annotations were carried out using an automated annotation server GhostKOALA^34^ with an e-value threshold of 1e-10. To further explore the specific pathways and biological functions, DEGs were mapped using MapMan (http://mapman.gabipd.org)^35^ tools. KEGG pathway enrichment analysis was conducted using KOBAS (http://kobas.cbi.pku.edu.cn/)^36^ and GO enrichment analyses was performed using Benjamini–Hochberg correction through AgriGO v2^37^. For transcription factor prediction, the transcripts were blasted against the transcription factors (TFs) of *Saccharum officinarum* (http://planttfdb.gao-lab.org/index.php?sp=Sof), identified from the protein dataset sourced from UniGene and PlantGDB. All methods were compiled according to relevant institutional, national, international guidelines and legislation.

## Results

### Early emergence of the SBRs in Erianthus wild germplasm

**Figure 1:**
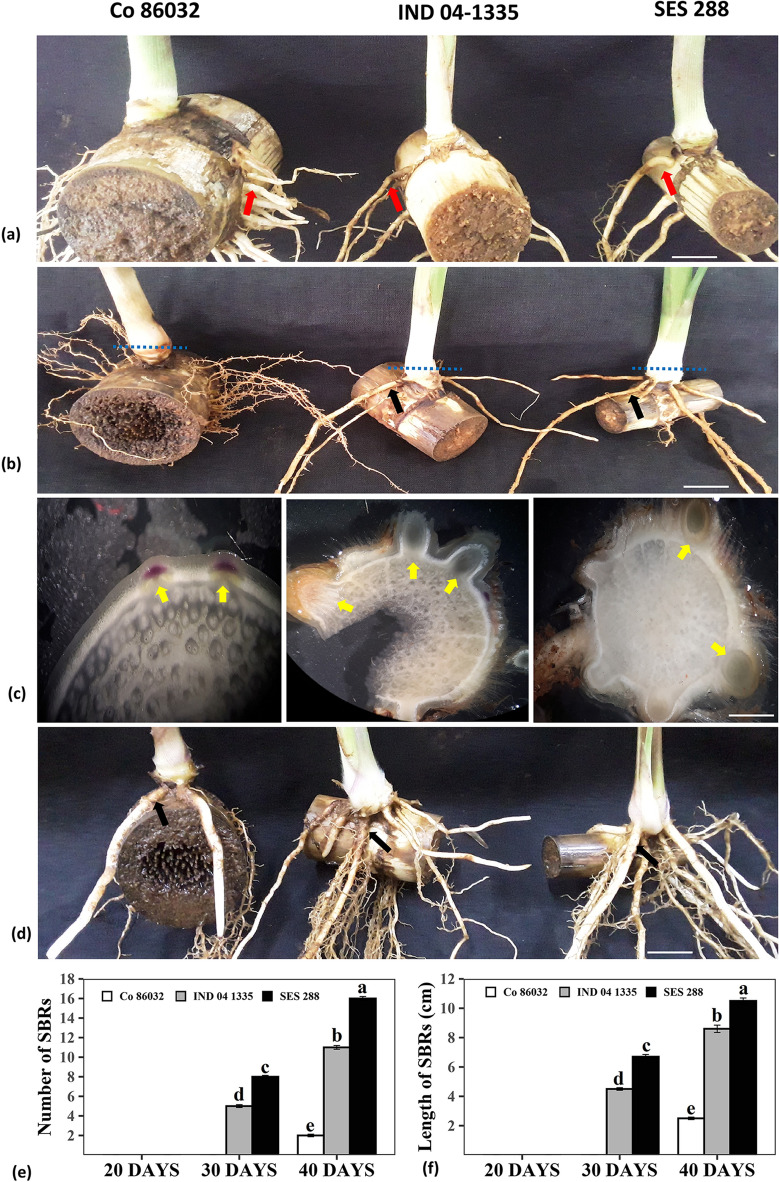
Sugarcane Shoot Borne Root (SBR) emergence and establishment at different stages of settling development in commercial hybrid Co 86032 and *Erianthus* clones (IND 04-1335, SES 288). (**a**) Roots phenotyped after 20 days of planting, (**b**) 30 days after planting. Scale bar 1 cm, (**c**) cross section of the shoot base. Scale bar 50 µm, (**d**) Root phenotyped after 40 days of planting. Scale bar 1 cm. The dotted lines shows the position of cross sections made to identify the emergence of SBR primordia. Red arrows indicate the sett roots, black arrows indicate the SBRs and blue arrows indicate the SBR primordial, (**e**) Number of shoot borne roots (Mean of ten replications), (**f**) length of shoot borne roots at 20, 30 and 40 DAP (Mean of ten replications). Different letters indicate significant difference at *p* < 0.05.

Root phenotyping, from 20 to 40 days of the settlings, indicated early emergence and establishment of large number of SBRs in *E. arundinaceus* clones (IND 04-1335 and SES 288) compared to the SBRs of commercial variety Co 86032. Phenotyping at 20 DAP showed only the formation of sett roots in both *Erianthus* and the commercial variety Co 86032 (Fig. 1a). At 30 DAP, the establishment of the SBRs was observed in both the *Erianthus* clones, while Co 86032 did not show any visible SBRs (Fig. 1b). The anatomical section of the shoot node at 30 DAP showed established SBRs in both the *Erianthus* clones whereas the root primordia got just initiated in case of Co 86032 (Fig. 1c). Only two SBRs of 2.5 cm length were observed in Co 86032 at 40 DAP whereas both the *Erianthus* clones showed completely established 11-16 SBRs of 8 to 10 cm length (Fig. 1d–f).

### Drought adaptive morphological and anatomical traits in SBRs

**Figure 2:**
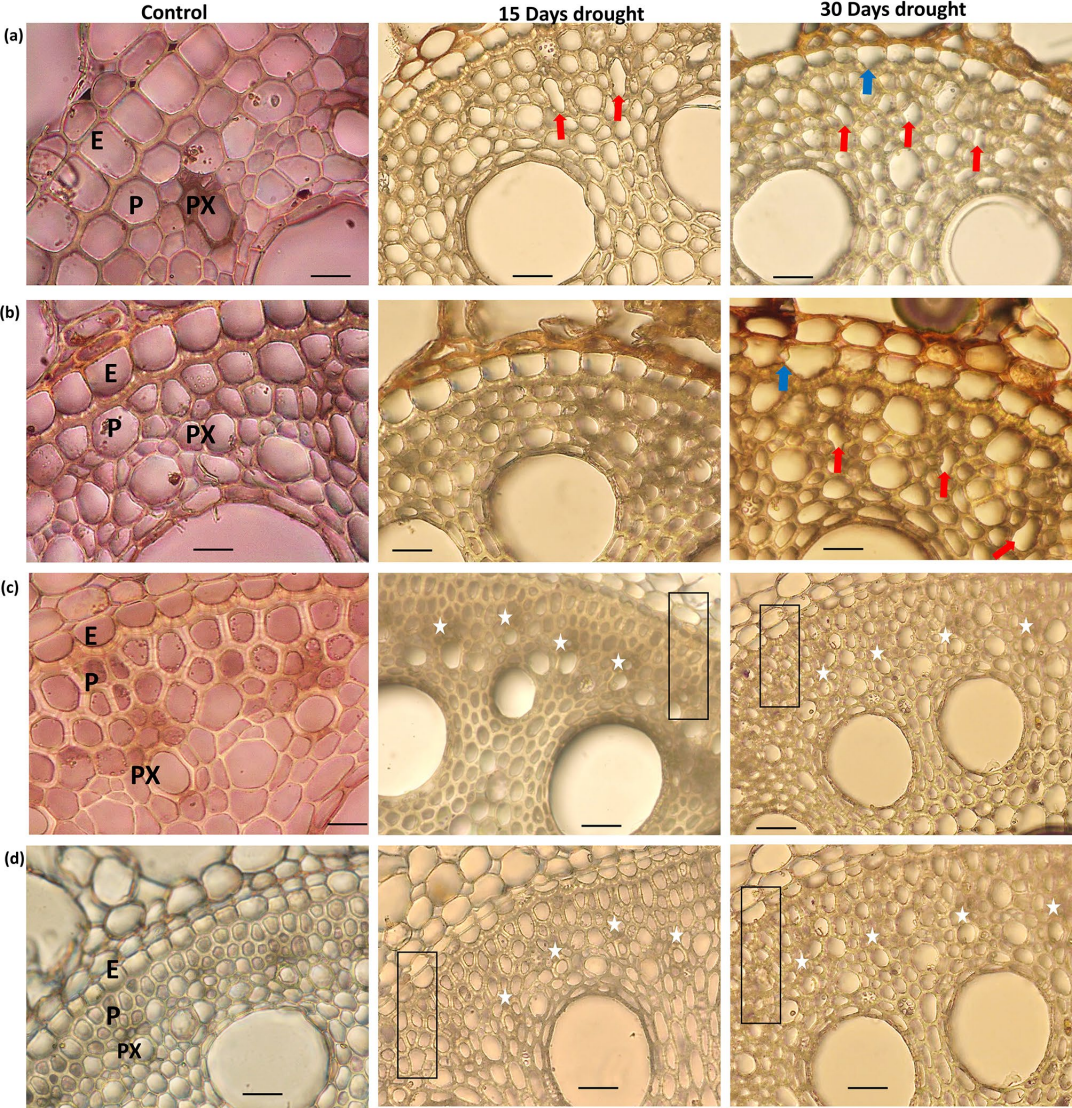
Anatomy of SBRs under control and at 15 and 30 days after drought exposure of commercial hybrids (Co 775, Co 86032) and *Erianthus* clones (IND 04-1335, SES 288). (**a**) Co 775, (**b**) Co 86032, (**c**) IND 04-1335, (**d**) SES 288. E-Endodermis, P-Pericycle, PX-Protoxylem. Red arrows indicate the collapsed protoxylem in Co 775 and Co 86032. Blue arrows indicate the collapsed endoderm layer, asterisks indicate the intact protoxylem elements and rectangular boxes highlight the cell wall thickening around endodermis, pericycle and protoxylem in IND 04-1335 and SES 288. Scale bar = 100 µM.

In order to study the morphological and anatomical traits of SBRs under drought, two *E. arundinaceus* (IND 04-1335 and SES 288) clones and two commercial varieties such as Co 775 (drought susceptible check) and Co 86032 (popular variety) were subjected to drought stress at 45 DAP. Before imposing stress the soil moisture content at field capacity was found to be 15.13% and the permanent wilting point was 6.34%. After 15 days of drought exposure, visible leaf rolling and drying symptoms were observed in the commercial clones, while *Erianthus* clones did not show any phenotypic symptoms of drought stress (Fig. S1a,b). At this stage, the soil moisture content in the drought imposed pots was found to be 50% of the field capacity, while the control pots were maintained at 100% field capacity. Both biomass and the length of SBRs were found to be significantly reduced after 15 days of exposure to drought stress in both the commercial varieties (Fig. S1c,d). The susceptible commercial clone Co 775 showed severe drying and browning of the SBRs compared to that of other commercial clone Co 86032 as well as *Erianthus* clones (Fig. S1b). The transverse sections of the SBRs showed significant anatomical variations under drought conditions among *Erianthus* and commercial clones, after 15 days of drought exposure. Under drought conditions, though significant thickening of the cell walls was visible around the endodermis, pericycle and protoxylem poles in both the *Erianthus* clones, cell wall thickening was highly prominent in IND 04-1335 (Fig. 2a–d). There was no prominent cell wall thickening found in Co 775 and Co 86032 under drought stress conditions. The protoxylem elements in IND 04-1335, SES 288 and Co 86032 were found to be intact, while the susceptible commercial clone Co 775 showed deformed protoxylem elements.

**Figure 3:**
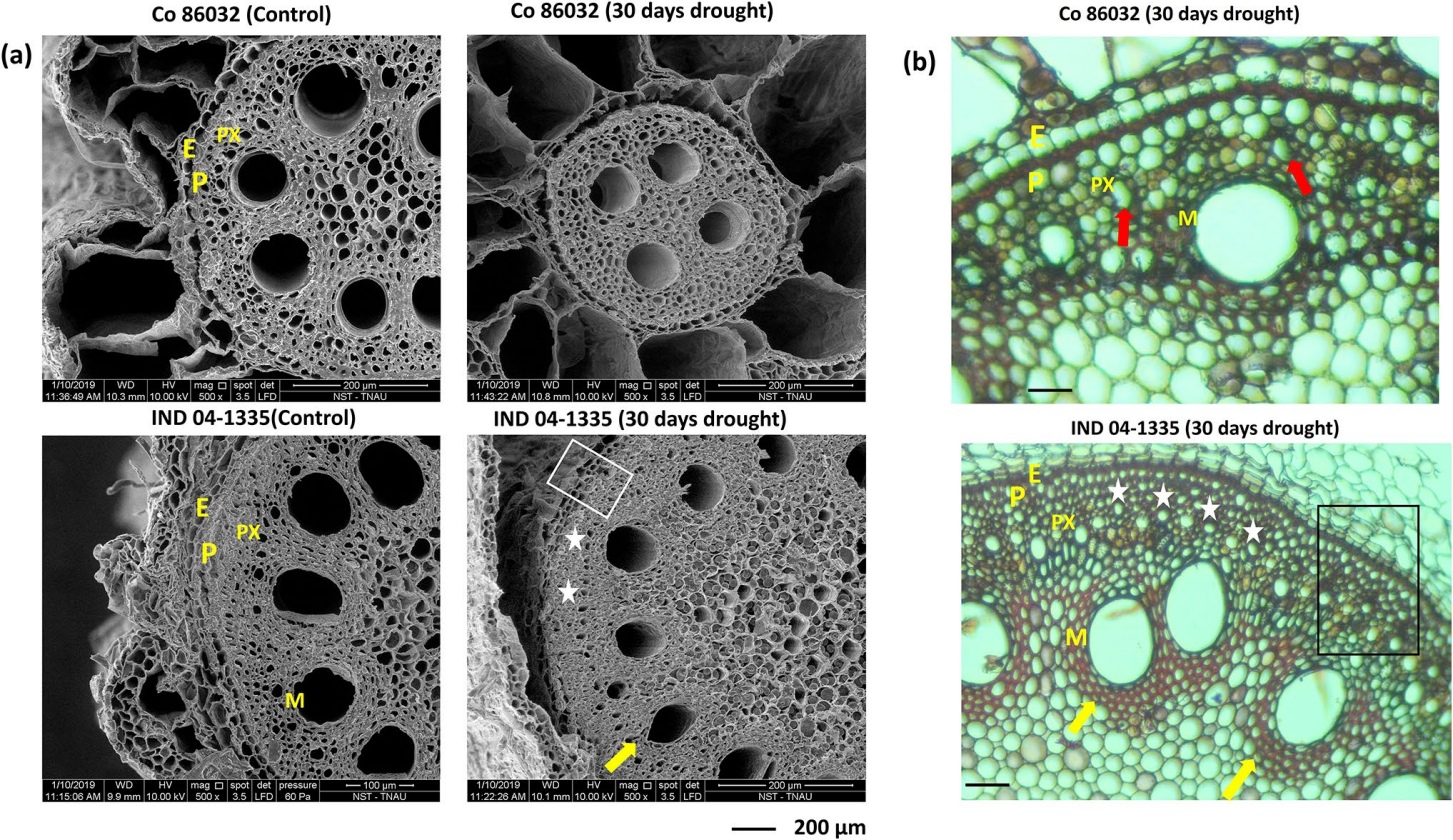
(**a**) Scanning Electron Micrographs of shoot borne roots at 30 days after drought exposure. (**b**) Root sections stained with phloroglucinol-HCl stain. Scale bar = 100 µM, yellow arrows indicate the reduced metaxylem diameter with cell wall thickening in IND 04-1335, red arrows indicate the deformed protoxylem in Co 86032, asterisks indicate the intact protoxylem elements and rectangular box indicate the lignified cells around endodermis, pericycle and protoxylem. E-Endodermis, P-Pericycle, PX-Protoxylem, M-Metaxylem.

After 30 days of drought exposure, the soil moisture content was measured at 30% of the field capacity. In this stage, the shoots of both the commercial clones showed severe drying symptoms whereas the *Erianthus* clones still maintained green and turgid leaves (Fig. S2). The anatomical observations of the SBRs showed three significant variations between the *Erianthus* and commercial clones (Fig. 2a–d). Both Co 775 and Co 86032 exhibited severely deformed protoxylem poles and the degradation of endodermis cell wall, while these cell layers were found to be intact in both the *Erianthus* clones (Fig. 2a–d). In addition to the thickening of cell walls below the endodermis, pericycle and xylem poles, a significant reduction was observed in metaxylem diameter in both *Erianthus* clones after 30 days of drought exposure (Fig. 3a,b).

### Transcriptome data generated from SBRs

A comparative transcriptome analysis was conducted between the drought-stressed and control SBR samples of IND 04-1335 and Co 86032. The transcriptome sequencing of the SBR samples from IND 04-1335 (control and 15 days drought stressed conditions respectively), generated 176 and 162 million raw reads, while 175 and 147 million raw reads were obtained from Co 86032 under the same conditions (Table 1). Quality trimmed sequences with a Phred score greater than 20 (Q20) resulted in 171, 157, 170 and 143 million reads from the control and drought-stressed IND 04-1335 and Co 86032 SBR samples correspondingly. The high quality reads of control and stressed SBR samples of both IND 04-1335 and Co 86032 were combined and assembled. The assembled transcript showed 581596 contigs in IND 04-1335 with an N50 length of 1382 bp, while it was 546074 contigs in Co 86032 with an N50 length of 1379 bp (Table S1). The assembled data for the unigenes showed 352796 contigs with an N50 length of 752 in IND 04-1335, while in case of Co 86032, it was 339128 contigs with an N50 length of 718. Completeness of the assembly were assessed using BUSCO analyses method which found about 70 to 77% completeness in the assembly (Table S2). The analyses showed minimum missing and fragmented BUSCOs.

**Table 1:** Data quality summary of the whole transcriptome generated from the shoot borne roots of Co 86032 and IND 04-1335, C-Control, S-Stress.

Samples	Raw reads	Clean reads	Error (%)	Q20 (%)	GC (%)
IND 04 1335 C	176.705358	171.957348	0.02	98.14	55.80
IND 04 1335 S	162.189068	157.953130	0.02	98.24	55.35
Co 86032 C	175.946796	170.262694	0.02	97.98	51.07
Co 86032 S	147.346186	143.345824	0.02	98.14	54.86

### Transcript quantification and differential gene expression analysis

At FDR < 0.01, a total of 7831 DEGs was identified in IND 04-1335 among which 3333 DEGs were found to be down-regulated whereas 4498 DEGs were found to be upregulated under drought condition. In Co 86032, a total of 9815 DEGs was identified in which 7106 DEGs were found to be down-regulated and 2709 DEGs were identified to be upregulated under drought condition. Among the upregulated transcripts in IND 04-1335, 85 DEGs showed log_2_ FC ≤ 20, 160 DEGs were with log_2_ FC ≤ 10 and 1400 DEGs showed log_2_ FC ≤ 5. In Co 86032, 36 DEGs showed log_2_ FC ≤ 10 and 300 DEGs showed log_2_ FC ≤ 5. The percentage of the downregulated DEGs was found to be higher in Co 86032 compared to IND 04-1335. The overview of the DEGs was generated as MA plot and heat map using the FDR-adjusted *p*-value < 0.01 for both the samples under control and stress conditions as shown in Fig. S3.

### Functional annotation of the differentially expressed genes

The initial annotation of the combined DEGs, against NCBI non-redundant (nr) database using the BLASTx program, showed 40% hit against *Sorghum bicolor* followed by *Zea mays*, *Setaria italica*, *Oryza sativa* and *Saccharum* hybrid (Fig. S4). The KOG classification of the DEGs showed the maximum number of transcripts in the class of secondary metabolite biosynthesis, cell wall membrane/envelope biogenesis, transport and catabolism in both IND 04-1335 and Co 86032 SBRs (Fig. S5).

In KEGG pathway enrichment analysis maximum number of differentially expressed transcripts from both IND 04-1335 and Co 86032 were annotated under biosynthesis of secondary metabolites pathway (Table S3 and S4). In IND 04-1335, metabolic pathways, biosynthesis of secondary metabolites, ribosomes, phenylpropanoid biosynthesis, carbon metabolism and glycolysis are the top 10 pathways that got enriched. In Co 86032, the biosynthesis of secondary metabolites, protein processing in endoplasmic reticulum, spliceosome and the biosynthesis of amino acids were among the top 10 KEGG-enriched pathways. GO annotations were obtained for 7350 DEGs of IND 04-1335 and 8370 DEGs of Co 86032. GO annotation showed annotated maximum number of transcripts under cellular component and biological process for both the samples (Fig. S6). Under the biological process, maximum number of genes from both the samples were annotated under cellular process, response to stimulus and the regulation of biological process. GO enrichment, carried out for the DEGs (FDR < 0.01) significantly upregulated in IND 04-1335 showed various enriched biological processes specifically involved in signalling drought stress, root growth and development (Table S5). IND 04-1335 upregulated transcripts showed enriched biological processes involved in positive regulation of the abscisic acid-activated signalling pathway (GO:0009789), MAPK cascade signalling under osmotic stress (GO:0005768), carbohydrate transport for lateral root development (GO:0005794), monosaccharide transmembrane transporter activity, root epidermal cell differentiation, auxin polar transport for root development (GO:0005634) and ethylene-activated signalling pathway for the regulation of post-embryonic root development (GO:0005789). About 38 transcripts that were significantly upregulated in IND 04-1335 were annotated under the enriched biological process ‘positive regulation of the response to water deprivation (GO:0005634). The GO enrichment analyses of the significantly-downregulated DEGs in Co 86032 showed the enriched processes involved in MAPK cascade signalling in response to osmotic stress, root meristem growth, positive regulation of the response to water deprivation, lateral root development, auxin-activated signaling pathway for lateral root development and ethylene-activated signalling pathway for the regulation of post-embryonic root development (Table S6).

**Figure 4:**
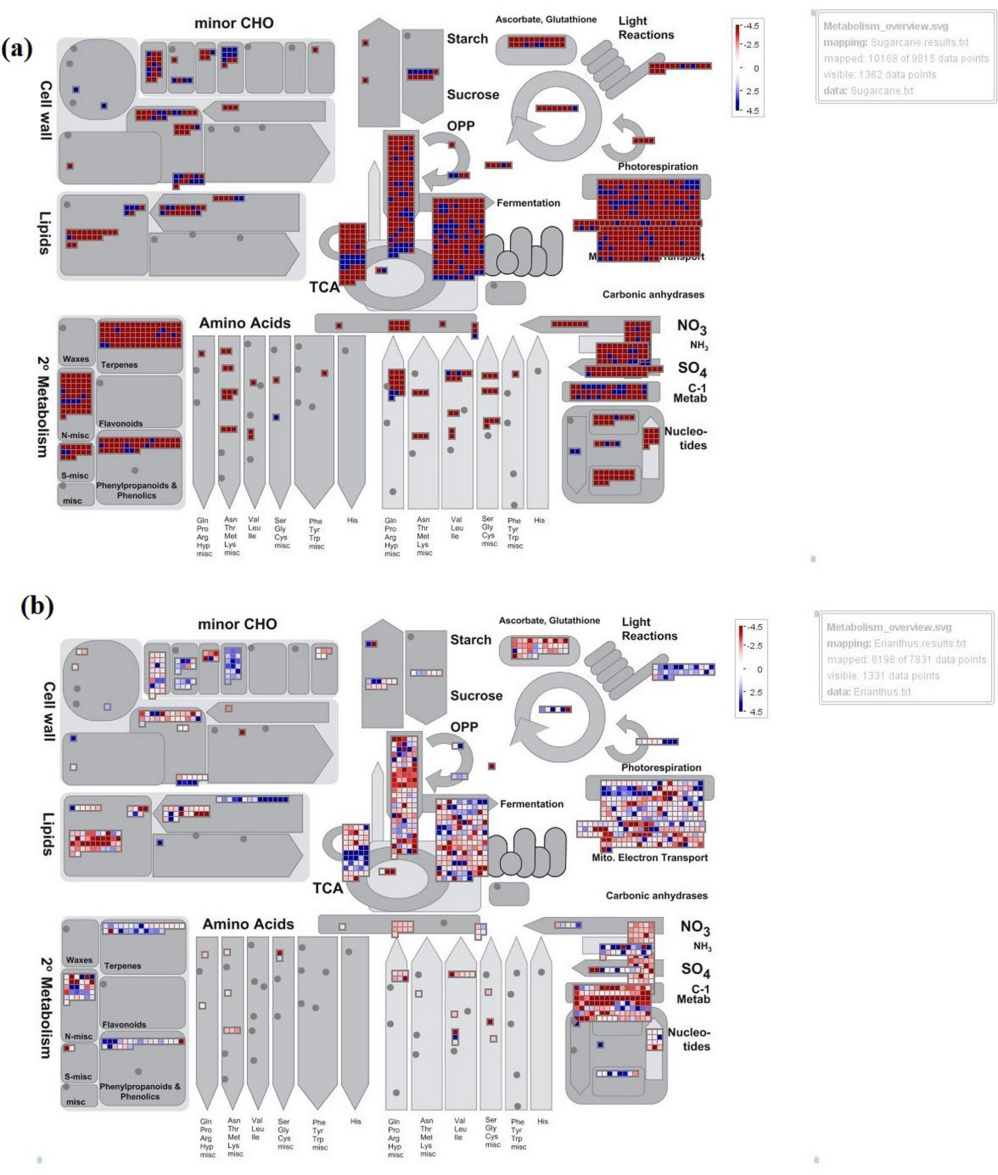
Metabolism overview of DEGs (FDR˂ 0.01) in IND 04-1335 and Co 86032 control and drought stressed samples. Transcripts showing more than log twofold difference between control and drought stressed samples have been mapped onto metabolism overview using MapMan software (http://mapman.gabipd.org/web/guest/mapman). Blue represents up-regulated genes, red represents down-regulated genes under drought stress condition, grey circles represent genes whose expression did not change more than twofold under drought stress condition. (**a**) Co 86032, (**b**) IND 04-1335.

MapMan analyses of the DEGs showed a significant upregulation of the transcripts that are involved in overall metabolism of IND 04-1335 compared to Co 86032 (Fig. 4). In Co 86032, a significant down regulation was observed for several transcripts involved in the synthesis of minor carbohydrates, lipids, secondary metabolites and photorespiration. Based on the enrichment analyses, the differential expression of specific transcripts involved in drought regulation and signalling among IND 04-1335 and Co 86032 are detailed in the following sections (Supplementary data E1).

### Higher abundance of transcripts involved in the synthesis of sugars and cell wall components in IND 04-1335

**Figure 5:**
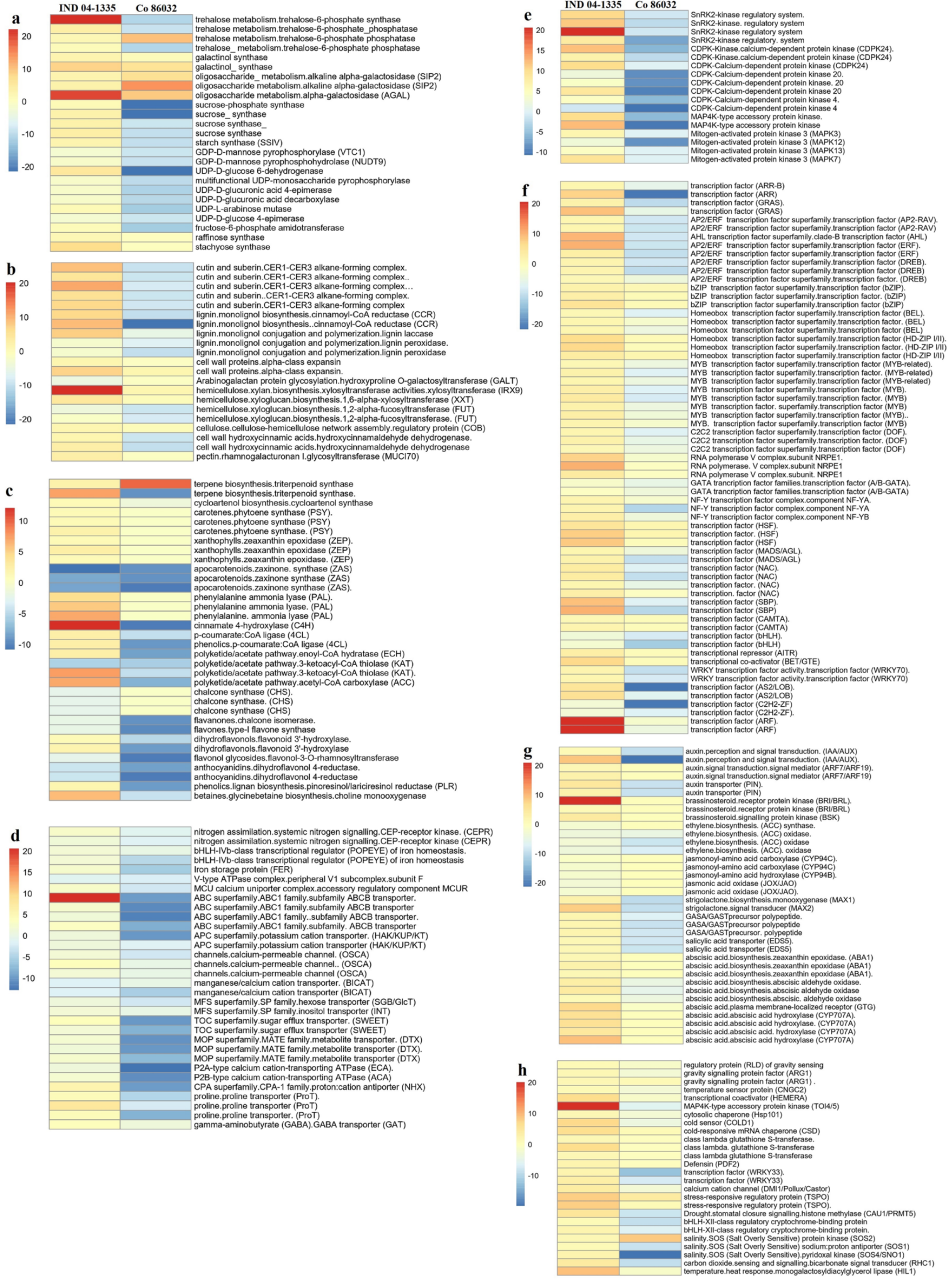
Heat map showing differentially expressed genes (DEGs) between IND 04-1335 and Co 86032 under drought condition. Different colors show the value of fold change, blue represent down-regulated genes and red represent up-regulated genes under drought stress condition. (**a**) DEGs involved in sugar synthesis, (**b**) DEGs involved in synthesis of cell wall components, (**c**) DEGs involved in secondary metabolite synthesis, (**d**) DEGs involved in metal and nutrient uptake, (**e**) Differentially expressed genes encoding for protein kinases, (**f**) DEGs encoding transcription factors, (**g**) DEGs involved in hormonal regulation and (**h**) Heat map of DEGs involved in signalling external stimuli.

Genes such as trehalose-6-phosphate synthase, trehalose-6-phosphate phosphatase, sucrose synthase, raffinose synthase and stachyose synthase involved in the synthesis of sugars exhibited higher abundance in IND 04-1335 roots (Fig. 5a). In this study, genes encoding for Alpha-galactosidase (SIP2, AGAL) showed an upregulation in both IND 04-1335 and Co 86032 under drought stress condition. About 145 transcripts in IND 04-1335 and 82 transcripts in Co 86032, involved in the synthesis of cell wall components, were found to be differentially regulated. The genes involved in the synthesis of cutin, suberin, lignin, xylan, arabinogalactan, and cell wall protein like expansin exhibited a significant upregulation in IND 04-1335 roots under drought stress (Fig. 5b). Several transcripts involved in the synthesis of cell wall hydroxycinnamic acids (hydroxycinnamaldehyde dehydrogenase) and xylan (xylosyltransferase: IRX9) were found to be upregulated in IND 04-1335 than Co 86032. On the other hand, the genes involved in the synthesis of xyloglucan (1,2-alpha-fucosyltransferase; FUT) showed down regulation in both IND 04-1335 and Co 86032, while the down regulation was too significant in Co 86032.

### Differential regulation of genes involved in the synthesis of secondary metabolites for root cell wall modification and antioxidation under drought stress

About 150 DEGs, involved in secondary metabolite biosynthesis for cell wall modification and antioxidation, were found to be differentially regulated in both IND 04-1335 and Co 86032 under drought stress. Most of the DEGs, involved in secondary metabolism, showed a high abundance in IND 04-1335 roots and a significant down regulation in Co 86032 (Fig. 5c; Fig. S7). Several transcripts of Phenylalanine Ammonia Lyase (PAL) showed higher abundance in IND 04-1335 under drought condition. Further, numerous transcripts involved in the biosynthesis of carotenoids, triterpenoids, flavones, dihyroflavonols, anthocyanidins, phenolics, phenlypropanoids, lignin, lignan, cutin and suberin through phenylpropanoid pathway were found to be significantly upregulated in the roots of IND 04-1335. Those genes involved in the biosynthesis of apocarotenoids (Zaxinone Synthase (ZAS)) and chalcones (Chalcone Synthase (CHS)) showed a significant down regulation in both the genotypes under drought condition. Choline monooxygenase, involved in the biosynthesis of glycine betaine, showed four fold upregulation in the roots of IND 04-1335 (Fig. 5c; Fig. S7).

### Significant upregulation of metal and nutrient transporters in IND 04-1335 under drought condition

The SBRs of IND 04-1335 exhibited a significant upregulation of genes involved in the transport of calcium, iron, potassium and other nutrients (Fig. 5d). Calcium transporters like calcium-permeable channel (OSCA), manganese/calcium cation transporter (BICAT), P2A/P2B-type calcium cation-transporting ATPase and MCU calcium uniporter complex were found to be significantly upregulated in IND 04-1335 while they were significantly down regulated in Co 86032. The genes such as ABC family transporters and APC superfamily potassium cation transporters, hexose transporter, sugar efflux transporter (SWEET), proline and GABA transporter were found abundantly in IND 04-1335 (Fig. 5d).

### Differential upregulation of important protein kinases and key transcription factors involved in drought stress signalling in IND 04-1335

Several transcripts that encode protein kinases viz., sucrose nonfermenting-related protein kinase 2 (SnRK2) complex, calcium-dependent protein kinases (CDPK4, CDPK20 and, CDPK 4, CDPK 4) and Mitogen-activated protein kinases (MAPK3, MAPK7, MAPK12, MAPK13) showed ten fold upregulation in IND 04-1335 (Fig. 5e). About 453 transcription factors (TFs) showed a differential regulation in the SBRs of IND 04-1335 whereas 253 TFs were differentially regulated in Co 86032 under drought stress conditions. Some of the key TFs viz., NAC, AP2/ERF superfamily, MYB, MYB related, bZIP, NF-YA, WRKY and MADS that get induced through various signalling pathways (CDPK, MAPK, SnRK2) were found to be significantly abundant in the roots of IND 04-1335 (Fig. 5f). The transcripts that encode for *SQUAMOSA* promoter binding protein (SBP), GRAS family genes, TFs involved in ethylene signalling (AP2/ERF-Ethylene Response Factors, DREB-Dehydration Responsive Element Binding factor) and auxin signalling (ARF-Auxin Response Factors) showed up to five times upregulation in IND 04-1335. TFs like AHL (AT-hook motif nuclear-localized) showed more than ten-fold upregulation and AS2/LOB (Lateral Organ Boundaries) showed up to four times upregulationin IND 04-1335, while they showed significant down regulation in Co 86032. The transcripts of WRKY70, Homeobox (HD-ZIP I/II), HSF (Heat Stress Transcription Factors) and the transcripts of bZIP family showed four-fold upregulation in IND 04-1335 compared to a mere 0.5- to 1.5-fold upregulation in Co 86032. TFs viz., bHLH, C2H2-ZF and GATA family were found to be down regulated in the roots of both IND 04-1335 and Co 86032 under drought conditions. Majority of the TFs were found to be significantly down regulated in Co 86032 (Fig. 5f).

### Differential expression of the phytohormones in the roots of IND 04-1335 and Co 86032 under drought condition

In order to understand the role of phytohormone regulation under drought conditions, the expression pattern of genes involved in the biosynthesis of abscisic acid (ABA), salicylic acid, jasmonic acid, ethylene, brassinosteriods, strigolactone and auxin signalling was analysed. Several genes that regulate ABA biosynthesis and signalling (AAO, ZEP/ABA1, CYP707A) and auxin transport/signalling (PIN, ARF7/ARF19, IAA/AUX) were found to be abundantly present in IND 04-1335 roots (Fig. 5g). Brassinosteroid signalling protein kinase (BSK), Brassinosteroid receptor protein kinase, GASA-precursor polypeptide and More Axillary Branching genes (MAX1 and MAX2) involved in strigolactone biosynthesis were found to be upregulated up to four times in IND 04-1335 whereas, they were significantly downregulated in Co 86032 (Fig. 5g). Those genes involved in ethylene biosynthesis (ACC synthase and ACC oxidase) and jasmonic acid pathway (JOX/JOA, CYP94C/CYP94B) were found to be significantly downregulated in both IND 04-1335 and Co 86032 roots under drought condition.

### Differential regulation of the transcripts involved in the signalling of external stimuli

The differential regulation of the transcripts that are specifically involved in signalling and regulation of the external stimuli under abiotic stresses were identified among the DEGs (Fig. 5h). About 70 DEGs in IND 04-1335 and 40 DEGs in Co 86032 involved in the regulation of external stimuli showed differential expression. Histone methylase (CAU1/PRMT5), glutathione S-transferase, bHLH-XII-class regulatory protein, WRKY33 transcription factor, Salt Overly Sensitive genes (SOS1/SOS2, SOS4/SNO1), stress-responsive regulatory protein (TSPO), monogalactosyldiacylglycerol lipase (HIL1), MAP4K-type accessory protein kinase (TOI4/5) and cytosolic chaperone (Hsp101) were found to be significantly upregulated in the roots of IND 04-1335 under drought conditions.

## Discussion

Sugarcane, a commercial crop, is planted from vegetative setts and after harvesting, it is ratooned over multiple years^22^. Sugarcane settlings develop sett roots during germination while in later stages, it develops a permanent root system called Shoot Borne Roots (SBR)^14^. Healthy SBRs significantly contribute to biomass production and drought tolerance in sugarcane^15^. A sugarcane variety with a better root system not only contributes to biomass yield, also tends to resist the lodging of the crop which is a desirable trait for sugarcane mechanical harvesting^38^. Due to the key impact of SBRs upon yield and adult fitness in maize, the developmental and molecular functions of maize SBRs have been studied extensively as an important breeding target^39–41^. However, the studies focusing on sugarcane SBRs are limited while its functional role remains largely unknown. So, the current study provides important insights about drought-adaptive plasticity and transcriptomics of the sugarcane SBRs.

The initial phenotyping of sugarcane wild germplasm *Erianthus* (IND 04-1335 and SES 288) clones showed early emergence and the establishment of SBRs. In contrast to the earlier studies in which SBRs have been reported to emerge within 5–7 days of sett planting the commercial sugarcane hybrids, Co 86032 showed a significant delay in the emergence of SBRs^14^. The present study indicated an early emergence as well as early conversion of sett roots to SBRs in *Erianthus*. Moisture stress has been reported to severely limit the productivity in sugarcane compared to the rest of the abiotic stresses^42^. Drought-adaptive plasticity in RSA helps in counteracting the limited water supply which ultimately improves the whole crop tolerance to drought stress^28^. The anatomy of the SBRs exhibited significant adaptation in IND 04-1335 under drought condition. The thickening of the cell walls around endodermis, pericycle and xylem poles, observed in *Erianthus* clones under drought stress, is considered to be a significant developmental plasticity to improve the hydraulic conductivity^43,44^. The metaxylem vessels tend to undergo cavitation and collapse under low water potential^45^. Cell wall thickening reduces the diameter of the metaxylem which in turn helps in reducing the cavitation pressure in order to maintain the conductivity under drought condition^45,46^. These scenarios i.e., reduced metaxylem diameter and cell wall thickening, observed in *Erianthus* establish its anatomical adaptation to protect the conducting tissues under drought. The thickening of cell walls, around the meristamatic pericycle layer, further indicates an adaptive trait to protect this layer from drought-induced damage, since this is the source for new lateral roots.

The comparative transcriptome analysis of SBRs generated superior data compared to the earlier transcriptome data generated among *Erianthus* and commercial hybrids for salinity stress^47^. The transcriptome of SBRs showed higher differential expression in IND 04-1335 compared to the commercial variety Co 86032. The pathway annotation and enrichment analyses also showed significant enrichment of pathways viz., abscisic acid-activated signalling, ethylene-activated signalling, auxin activated and MAPK cascade signalling in *Erianthus* clone IND 04-1335^12,13^. These pathways are reported to operate as important signal transduction pathways to mediate drought stress signalling^32,33^. Also an active transcriptional response of these signalling cascades helps in activating the expression of downstream resistance genes to impart drought tolerance^48–50^.

### Sugar signalling and cell wall modifications in IND 04-1335 SBRs to impart drought tolerance

Significant upregulation of the genes involved in the synthesis of sugars like trehalose, sucrose, raffinose and stachyose in IND 04-1335 roots, might be related to higher accumulation of these sugars under drought condition^11,51^. Sugars are the major nutrient/structural constituents of the cells, which act as stress signalling molecules to maintain the osmotic homeostasis under drought conditions^52^. The presence of abundant trehalose-6-phosphate synthase and trehalose-6-phosphate phosphatase in IND 04-1335 SBRs indicates a high accumulation of trehalose sugar. The over expression of trehalose-6-phosphate synthase and trehalose-6-phosphate phosphatase has been shown to enhance drought tolerance by stabilizing the cell structure under drought stress^11,53^. Cell wall modification in IND 04-1335 roots was evident from the significant upregulation of the genes involved in the synthesis of cellulose, hemicellulose, xylan, arabinogalactan, cutin, suberin, lignin and cell wall protein like expansin^54^. Heavy accumulation of the cell wall polymers like lignin, suberin and cutin in *Erinathus* might have resulted in cell wall thickening, as evidenced from the anatomical data^11,55^. Further, cell wall modification also functions as a protective barrier to prevent the conducting tissues from moisture stress damage ^23,55^.

Several studies infer the significant accumulation of secondary metabolites as an adaptive mechanism under drought condition. Upregulation of the secondary metabolites, involved in the biosynthesis of major phenlypropanoids, carotenoids, triterpenoids, flavones, dihyroflavonols, anthocyanidins, phenolics, lignin and lignan, was observed only in IND 04-1335. Phenylpropanoids are highly adaptable natural metabolites that are produced and accumulated in high levels to scavenge the reactive oxygen species (ROS) under stress conditions^15,56^. The abundant quantity of other metabolites like lignin and suberin in IND 04-1335 roots would have contributed towards strengthening the cell wall and reduction of the membrane lipid peroxidation under reduced moisture levels^57^.

### Calcium signalling and the expression of key metal transporters to enhance the drought tolerance in IND 04-1335 SBRs

Calcium ion (Ca^2+^) is reported to be an important component in mediating the stress responses in higher plants^58,59^. Cytosolic calcium levels are reported to be elevated during drought through the regulation of influx and efflux channels. The calcium levels serve as a signal as perceived by Ca^2+^ sensors and the signals are detected and transmitted by specific protein kinases like CDPK and MAPK to induce drought-responsive key transcription factors^60,61^. The abundant presence of calcium transporters like calcium-permeable channel (OSCA), manganese/calcium cation transporter (BICAT) and P2A/P2B-type calcium cation-transporting ATPase indicates an active calcium signalling-mediated stress tolerance response in IND 04-1335 roots. Similarly, the higher abundance of APC superfamily potassium cation transporters indicates a higher uptake of potassium in IND 04-1335. As a key element, potassium increases the stability of the cell membrane and is involved in the regulation of root morphology^62^. Both proline and GABA are two important osmoprotective metabolites that get accumulated in plants in response to ROS generation^63,64^. The significant upregulation of the Proline transporters (ProT) and GABA transporters indicate that these osmoprotectants are heavily accumulated to regulate the antioxidation process in IND 04-1335 roots.

### Significant up regulation of protein kinases and transcription factors in IND 04-1335 roots

Signal transduction by protein kinases such as Mitogen‐Activated Protein Kinase (MAPK) cascades, sucrose nonfermenting1 (SNF1) related protein kinases (SnRKs), and calcium‐dependent protein kinases (CDPKs) play a crucial role in cellular regulation and metabolism under stress conditions^54,58,65^. Targeted protein phosphorylation by protein kinases is an important plant stress response to regulate the downstream stress signalling pathways^58,66,67^. The abundant presence of transcripts that encode CDPK, MAPK and SnRK2 complexes shows an active protein kinase signalling pathway to induce a downstream drought-adaptive gene expression in IND 04-1335^66,68^.

A significant upregulation of the TFs viz., NAC, AP2/ERF superfamily, ARF, AHL, GRAS family, MYB, MYB related, bZIP, NF-YA, WRKY and MADS in IND 04-1335 infer their major role as a response to drought stress^11,69,70^. Further, the strong induction of ARF and AP2/ERF (APETALA2/ethylene responsive factor) in IND 04-1335 shows auxin- and ethylene-mediated stress tolerance response^60^. ARFs are also shown to promote root development under drought and saline stress conditions^11,71^. Similarly, the DREB TFs that belong to AP2/ERF family have been shown to regulate the downstream stress-related genes through dimerization with ERF-like TFs^13,72^. A significant upregulation of the WRKY70 TFs infers the promotion of root growth under stressful conditions by sustaining the ROS homeostasis^73^. More than ten-fold upregulation of the AHL transcription factor shows its strong functional response to drought in IND 04-1335. Under stress, the AHLs are reported to stop growth and development by inhibiting the growth-promoting Phytochrome-Interacting Factors (PIFs), thereby allowing the resources to be reallocated to stress-adaptive responses^74^. The upregulation of Squamosa promoter Binding Protein (SBP) family transcription factor in IND 04-1335 coincides with the earlier reports to impart tolerance upon drought and salinity^75^.

### Hormone signalling for enhanced drought tolerance in IND 04-1335 SBRs

Phytohormones are known to play an important role in mediating various physiological responses under drought conditions^76^. Among the major phytohormones, abscisic acid (ABA) and auxin-mediated stress sensing and signalling pathways have been proved to enhance stress tolerance in a variety of crops^77,78^. In the current study, several genes that regulate the ABA biosynthesis/signalling (AAO, ZEP/ABA1, CYP707A) and auxin transport/signalling (PIN, ARF7/ARF19, IAA/AUX) were found abundantly in IND 04-1335 SBRs. Increased ABA levels are also shown to induce the antioxidant enzyme activity and the deposition of suberin in roots to retain more water and enhance drought tolerance^79^. The higher expression of Brassinosteroid signalling protein kinase (BSK), Brassinosteroid receptor protein kinase (BRI/BRL) and Ethylene Responsive Factors shows the presence of ABA-independent stress tolerant pathway mechanism in IND 04-1335 roots^80^.

### Differential expression of the transcripts responding to stress stimuli

The transcriptome data showed differential expression of specific transcripts that are involved in signalling and the regulation of stress stimuli under abiotic stresses in IND 04-1335 and Co 86032 SBRs. Significant upregulation of specific regulatory proteins, chaperones, WRKY 33 transcription factor, glutathione S-transferases, Salt Overlay Sensitive genes (SOS1, SOS2 and SOS4) and Heat Inducible Monogalactosyldiacylglycerol lipase 1 (HIL1) may encode products that confer drought tolerance to IND 04-1335 roots. Among the WRKY TFs, WRKY33 has been reported to be the most intense transcription factor in response to drought stress. Similarly, glutathione *S*-transferases get induced by diverse abiotic stress and help in maintaining cell redox homeostasis, detoxification and antioxidant activity^81^. Heat Inducible Monogalactosyldiacylglycerol lipase (HIL1), a lipase gene significantly upregulated in IND 04-1335 roots, is reported to induce the catabolism of monogalactosyldiacylglycerol (MGDG) under heat stress in *Arabidopsis*^82^. This also indicates that the enzymes involved in lipid metabolism may play a role in imparting drought stress tolerance to IND 04-1335 roots^83^.

## Conclusion

**Figure 6:**
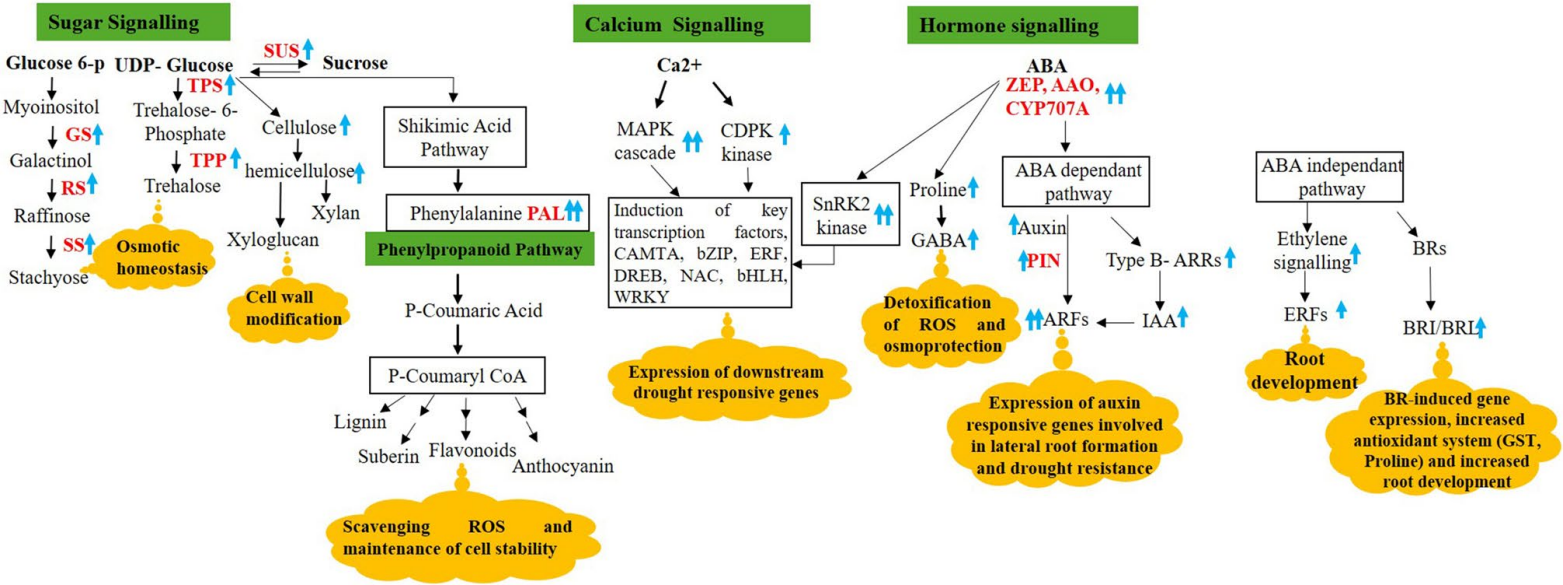
Overview of key genes/ molecular signaling pathways induced in higher abundance in SBRs of *Erianthus* (IND 04-1335) contributing to drought tolerance. The major signaling pathways are highlighted in green, the functional molecular and physiological mechanisms are highlighted in yellow. Blue arrows indicate up-regulation, two blue arrows indicate more than one gene and specific enzymes upregulated in different pathways are given in red font.

The current study indicates a healthy SBR system prevailing in *Erianthus* germplasm compared to the commercial hybrid Co 86032. The study also identified a delayed emergence and a poor SBR system in the commercial hybrid, Co 86032. Most of the anatomical traits (i.e., endodermal cell wall thickening, reduced metaxylem diameter, stele elements and pericycle layer protected with lignified cells), observed in *Erianthus*, were found to be altered under drought conditions. This scenario infers its drought-adaptive plasticity, which may significantly contribute to enhance the water uptake efficiency in *Erianthus*. While SBRs in the commercial hybrids Co 86032 and Co 775 showed high susceptibility to drought stress. The transcriptome data of the SBRs, under drought, coincides with that of the anatomical plasticity of the SBRs in *Erianthus*. The SBRs of the drought tolerant *Erianthus* clone (IND 04-1335) showed a significant upregulation of the genes that regulate four important stress-responsive signalling pathways viz., sugar, calcium and hormone signalling as well as phenylpropanoid biosynthesis (Fig. 6). Through these key signalling pathways stress-responsive physiological and biochemical mechanisms were triggered to impart drought tolerance in *Erianthus* SBRs. The current study is a first-of-its-kind report that unravel the morphological and molecular responses of sugarcane SBR under drought stress and provides a valuable reference for breeding drought tolerant sugarcane genotypes with better SBRs.

### Supplementary Information

Supplementary Figure 1.

Supplementary Figure 2.

Supplementary Figure 3.

Supplementary Figure 4.

Supplementary Figure 5.

Supplementary Figure 6.

Supplementary Figure 7.

Supplementary Information 8.

Supplementary Tables.

## Data Availability

The datasets generated and/or analysed during the current study are available in the NCBI-SRA database (PRJNA937025).
