# Supplementary figures and images for: Comparative transcriptome profiling to unravel the key molecular signalling pathways and drought adaptive plasticity in shoot borne root system of sugarcane
Source: Sci Rep. 2023 Aug 8;13:12853. doi: 10.1038/s41598-023-39970-1 (PMC10409851; doi:10.1038/s41598-023-39970-1)

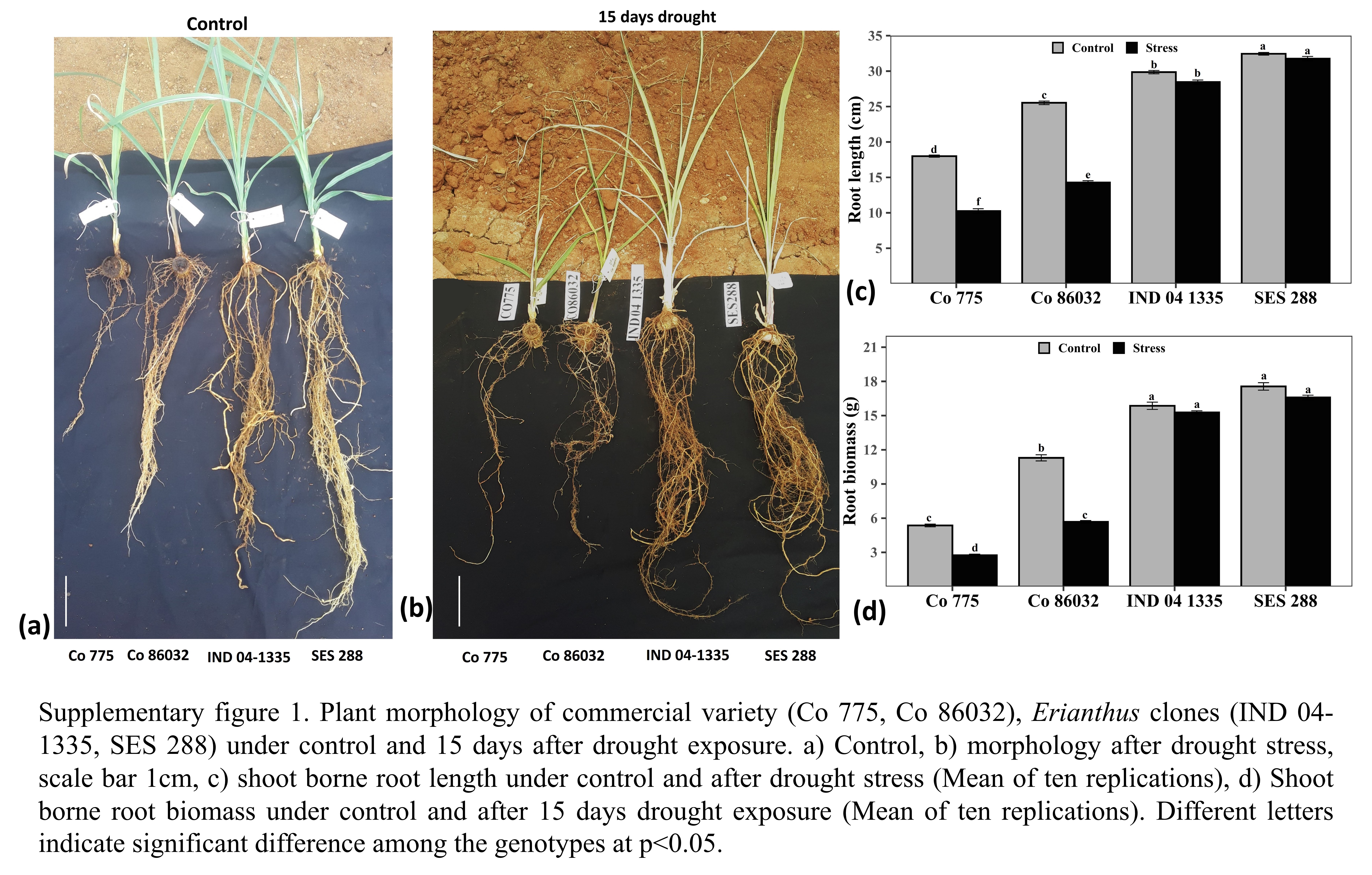

Supplement: Supplementary file 1 — Supplementary Figure 1. [file 41598_2023_39970_MOESM1_ESM.jpg]

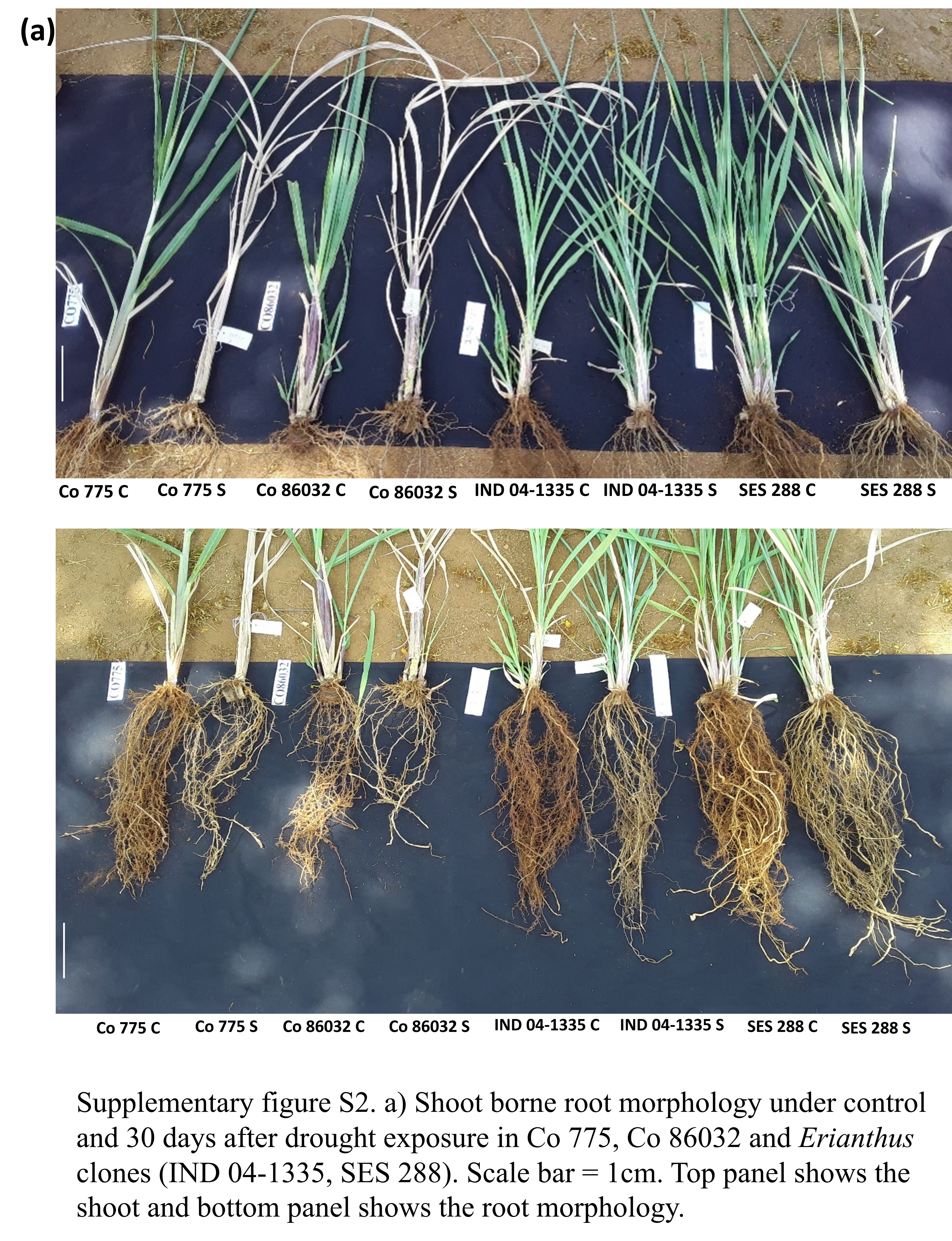

Supplement: Supplementary file 2 — Supplementary Figure 2. [file 41598_2023_39970_MOESM2_ESM.jpg]

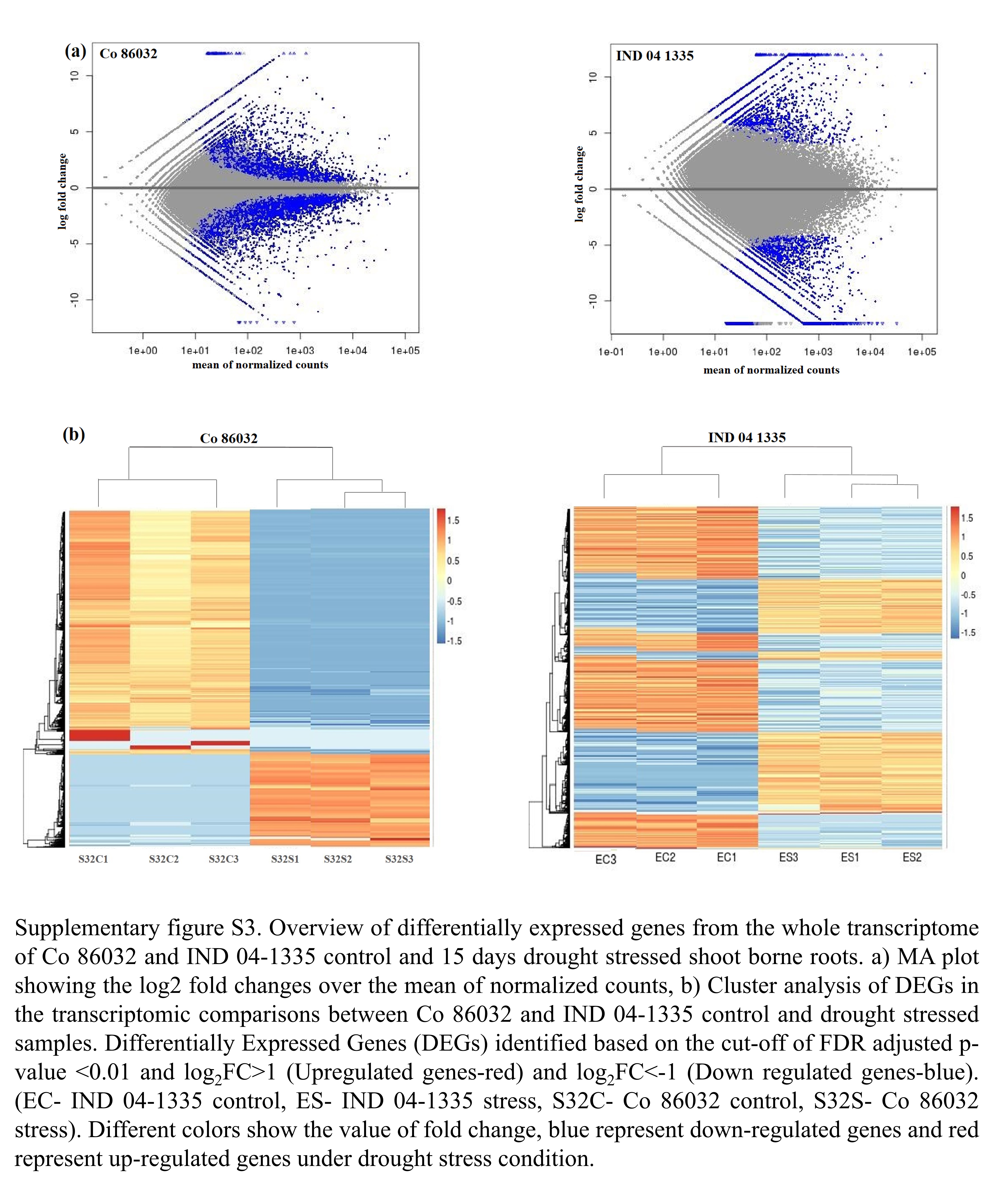

Supplement: Supplementary file 3 — Supplementary Figure 3. [file 41598_2023_39970_MOESM3_ESM.jpg]

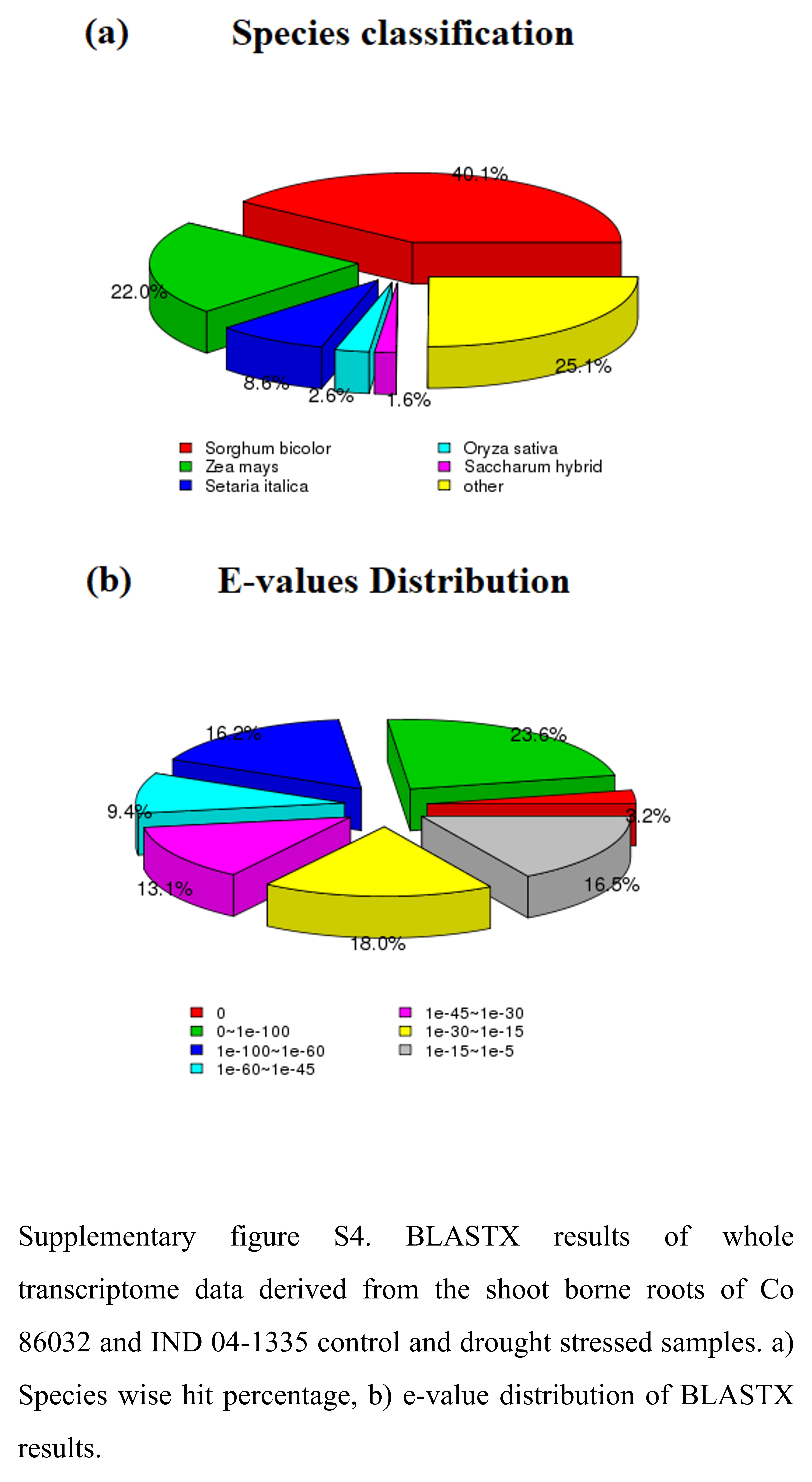

Supplement: Supplementary file 4 — Supplementary Figure 4. [file 41598_2023_39970_MOESM4_ESM.jpg]

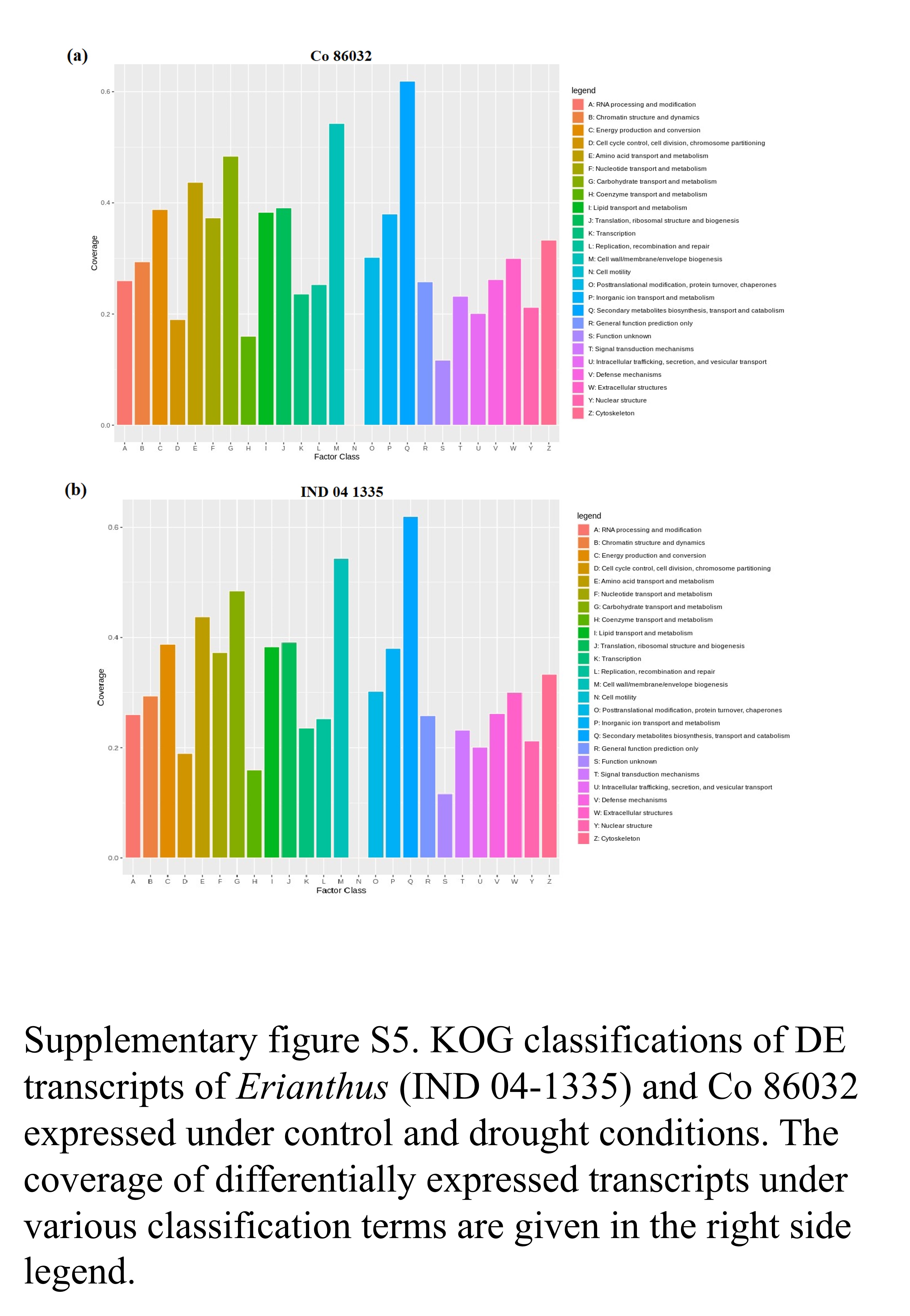

Supplement: Supplementary file 5 — Supplementary Figure 5. [file 41598_2023_39970_MOESM5_ESM.jpg]

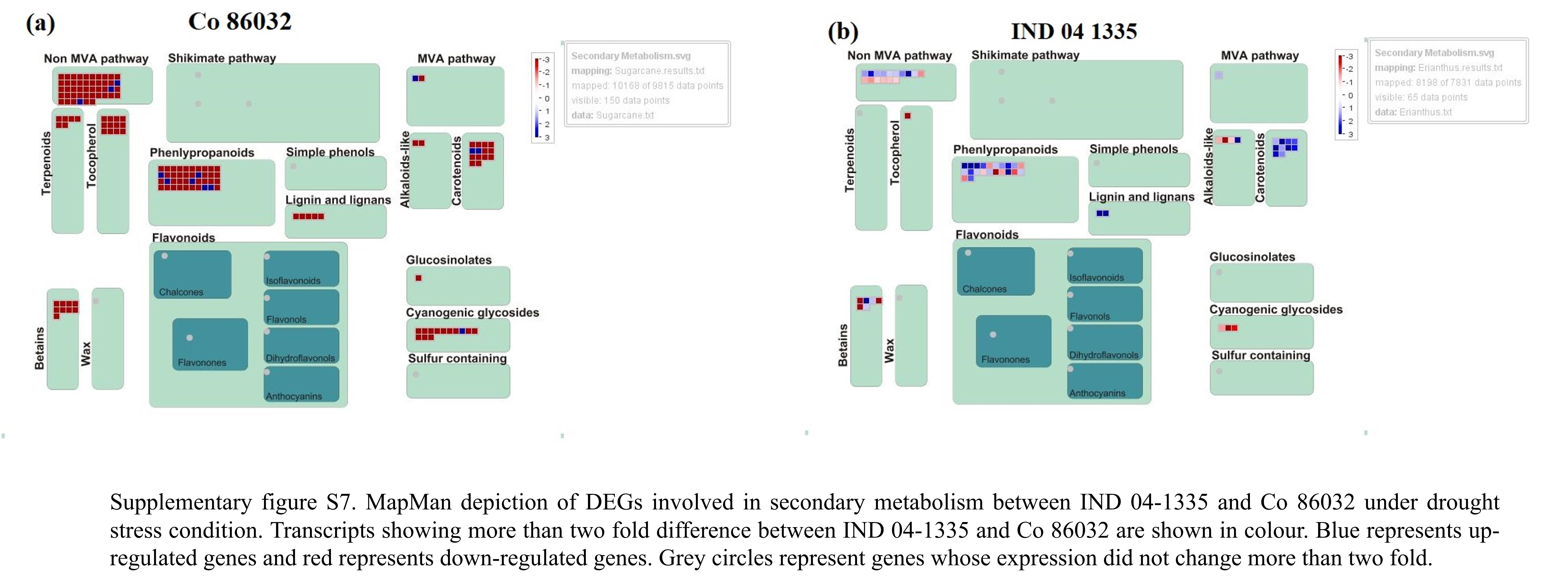

Supplement: Supplementary file 7 — Supplementary Figure 7. [file 41598_2023_39970_MOESM7_ESM.jpg]
