# Supplementary material for: Comparative transcriptome profiling to unravel the key molecular signalling pathways and drought adaptive plasticity in shoot borne root system of sugarcane
Source: Sci Rep. 2023 Aug 8;13:12853. doi: 10.1038/s41598-023-39970-1 (PMC10409851; doi:10.1038/s41598-023-39970-1)
Supplement: Supplementary file 9 — Supplementary Tables. [file 41598_2023_39970_MOESM9_ESM.docx]

**Table S1. Summary statistics of de novo assembled transcriptome data obtained from the shoot borne roots of IND 04-1335 and Co 86032**

|  | ***de novo* assembled transcriptome** | | | |
| --- | --- | --- | --- | --- |
|  | **IND 04-1335** | | **Co 86032** | |
| **Counts** | **Er_Trinity.fasta** | **Er_trinity_cdhit_s90c95.fasta** | **sac_Trinity.fasta** | **sac_trinity_cdhit_s90c95.fasta** |
| Total trinity 'unigenes' | 352796 | 352732 | 339128 | 339071 |
| Total trinity transcripts | 581596 | 521409 | 546074 | 492497 |
| Percent GC | 49.21 | 48.93 | 48.55 | 48.16 |
| **Stats based on ALL transcript contigs** |  |  |  |  |
| Contig N10 | 3907 | 3575 | 3626 | 3327 |
| Contig N20 | 2900 | 2591 | 2733 | 2460 |
| Contig N30 | 2264 | 1987 | 2175 | 1923 |
| Contig N40 | 1788 | 1532 | 1753 | 1513 |
| Contig N50 | 1382 | 1147 | 1379 | 1152 |
| Median contig length | 408 | 372 | 403 | 366 |
| Average contig | 782.82 | 693.64 | 775.36 | 686.57 |
| Total assembled bases | 455287112 | 361669266 | 423404922 | 338133252 |
| **Stats based on ONLY LONGEST ISOFORM per 'GENE'** | |  |  |  |
| Contig N10 | 3316 | 3314 | 3171 | 3170 |
| Contig N20 | 2177 | 2176 | 2177 | 2177 |
| Contig N30 | 1457 | 1456 | 1519 | 1518 |
| Contig N40 | 935 | 934 | 999 | 999 |
| Contig N50 | 630 | 630 | 661 | 660 |
| Median contig length | 300 | 300 | 292 | 292 |
| Average contig | 512.36 | 512.25 | 513.53 | 513.42 |
| Total assembled bases | 180757314 | 180686031 | 174151434 | 174084582 |
| **Stats based on Unigenes** |  |  |  |  |
| Contig N10 | 591 | 261 | 455 | 157 |
| Contig N20 | 723 | 415 | 556 | 283 |
| Contig N30 | 807 | 531 | 656 | 405 |
| Contig N40 | 853 | 598 | 754 | 514 |
| Contig N50 | 752 | 517 | 718 | 492 |
| Median contig length | 108 | 72 | 111 | 74 |
| Average contig | 270.46 | 181.39 | 261.83 | 173.15 |
| Total assembled bases | 274529798 | 180983235 | 249253488 | 164048670 |

**Table S2. BUSCO completeness analysis of the de novo assembled transcriptome data obtained from the shoot borne roots of IND 04-1335 and Co 86032**

| **Samples** |  | **Complete BUSCOs (C)** | **Complete and duplicated BUSCOs (D)** | **Fragmented BUSCOs (F)** | **Missing BUSCOs (M)** | **Total BUSCO groups searched** | **Completeness %** |
| --- | --- | --- | --- | --- | --- | --- | --- |
|  | **viridiplantae_odb10** |  |  |  |  |  |  |
| IND 04 1335 | C:99.1%[S:22.6%,D:76.5%],F:0.7%,M:0.2%,n:425 | 421 | 325 | 3 | 1 | 425 | 77.1 |
| Co 86032 | C:95.3%[S:26.6%,D:68.7%],F:3.1%,M:1.6%,n:425 | 405 | 292 | 13 | 7 | 425 | 72.0 |
|  | **poales_odb10** |  |  |  |  |  |  |
| IND 04 1335 | C:94.2%[S:21.4%,D:72.8%],F:2.0%,M:3.8%,n:4896 | 4613 | 3563 | 96 | 187 | 4896 | 77.2 |
| Co 86032 | C:92.0%[S:27.6%,D:64.4%],F:2.6%,M:5.4%,n:4896 | 4503 | 3154 | 128 | 265 | 4896 | 70.2 |

**Table S3. GO enriched biological process specifically involved in drought stress response in the upregulated genes of IND 04-1335 (FDR<0.01)**

| **Transcript ID** | **Gene Ontology annotation** | **FDR** |
| --- | --- | --- |
| TRINITY_DN4998_c0_g1 | GO:0005634, ethylene-activated signaling pathway, regulation of root development`response to water deprivation | 5.4169E-91 |
| TRINITY_DN9448_c0_g1 | GO:0031234, regulation of root morphogenesis | 7.63039E-64 |
| TRINITY_DN4922_c0_g1 | GO:0005730, lateral root development, regulation of response to water deprivation | 6.72666E-34 |
| TRINITY_DN10031_c1_g1 | GO:0005634,lateral root development` | 5.71088E-33 |
| TRINITY_DN58329_c0_g1 | GO:0016021, auxin-activated signaling pathway, lateral root development, response to water deprivation | 2.8803E-21 |
| TRINITY_DN859_c0_g1 | GO:0016021, response to water deprivation,root development | 1.07563E-17 |
| TRINITY_DN8671_c0_g1 | GO:0031901, root hair tip development | 1.41666E-17 |
| TRINITY_DN40_c0_g1 | GO:0005768, MAPK cascade`response to osmotic stress`root meristem growth` | 1.28595E-15 |
| TRINITY_DN347_c2_g1 | GO:0005634, positive regulation of response to water deprivation, | 4.52432E-15 |
| TRINITY_DN9092_c0_g1 | GO:0016021, regulation of root development, response to osmotic stress | 2.06416E-13 |
| TRINITY_DN30092_c0_g1 | GO:0005789, ethylene-activated signaling pathway, regulation of post-embryonic root development | 2.25084E-12 |
| TRINITY_DN5593_c1_g1 | GO:0005739, root development | 1.20416E-11 |
| TRINITY_DN3491_c0_g1 | GO:0033186, root meristem growth` | 2.56645E-11 |
| TRINITY_DN31435_c0_g1 | GO:0005634, lateral root development | 6.39283E-11 |
| TRINITY_DN2208_c0_g1 | GO:0016592, response to osmotic stress`root development | 7.88565E-09 |
| TRINITY_DN27537_c1_g1 | GO:0005829, auxin-activated signaling pathway`lateral root development` | 5.33748E-08 |
| TRINITY_DN6055_c0_g1 | GO:0005737, response to strigolactone, response to water deprivation | 7.4627E-08 |
| TRINITY_DN8178_c0_g1 | GO:0016021, regulation of root development,xylem and phloem pattern formation | 6.51256E-07 |
| TRINITY_DN11902_c0_g1 | GO:0005634, auxin polar transport`root development` | 1.13816E-06 |
| TRINITY_DN8334_c0_g2 | GO:0005737, maintenance of root meristem ,regulation of root development | 9.48901E-05 |
| TRINITY_DN1093_c0_g1 | GO:0005789,ethylene-activated signaling pathway, regulation of post-embryonic root development | 9.64983E-05 |
| TRINITY_DN23014_c0_g1 | GO:0005634, regulation of root development`response to chitin | 9.73859E-05 |
| TRINITY_DN2088_c0_g1 | GO:0005634, transcription cis-regulatory region binding`root hair cell development | 9.85089E-05 |
| TRINITY_DN34944_c0_g1 | GO:0043231, root meristem growth | 0.000104783 |
| TRINITY_DN12542_c0_g1 | GO:0016021, potassium ion antiporter activity, response to water deprivation, root development | 0.000108459 |
| TRINITY_DN12558_c0_g1 | GO:0009330, root development` | 0.000109623 |
| TRINITY_DN29048_c0_g1 | GO:0005886, root development | 0.000111779 |
| TRINITY_DN18435_c0_g2 | GO:0005634, auxin-activated signaling pathway`lateral root development | 0.000113011 |
| TRINITY_DN2369_c0_g1 | GO:0031225, root epidermal cell differentiation`root hair cell differentiation | 0.000114601 |
| TRINITY_DN47737_c0_g1 | GO:0005789, ethylene-activated signaling pathway, regulation of post-embryonic root development` | 0.000114641 |
| TRINITY_DN1743_c0_g1 | GO:0009507, lateral root development`,response to salt stress` | 0.000115068 |
| TRINITY_DN9077_c0_g1 | GO:0009789, positive regulation of abscisic acid-activated signaling pathway | 0.000116588 |
| TRINITY_DN1216_c0_g1 | GO:0005783, root hair cell development | 0.000117519 |
| TRINITY_DN5499_c0_g1 | GO:0005794, carbohydrate transport,lateral root development | 0.00018487 |
| TRINITY_DN2365_c1_g1 | GO:0005634, lateral root development | 0.000356712 |
| TRINITY_DN1113_c0_g1 | GO:0005634,positive regulation of response to water deprivation, root development | 0.000642997 |

**Table S4. GO enriched biological process specifically involved in drought stress response in the downregulated genes of Co 86032 (FDR<0.01)**

| #gene_id | **Gene Ontology annotation** | **FDR** |
| --- | --- | --- |
| TRINITY_DN4663_c0_g1 | GO:0005783, root development | 3.69E-07 |
| TRINITY_DN16_c0_g1 | GO:0005634,regulation of root meristem growth | 2.34E-06 |
| TRINITY_DN4636_c0_g1 | GO:0005634, maintenance of root meristem identity` | 3.98E-05 |
| TRINITY_DN0_c0_g1 | GO:0005737,maintenance of root meristem identity | 8.23E-05 |
| TRINITY_DN14_c0_g1 | GO:0016324,root hair initiation | 8.51E-05 |
| TRINITY_DN4608_c0_g2 | GO:0005794,root hair cell development | 8.51E-05 |
| TRINITY_DN4694_c0_g1 | GO:0009524,lateral root formation | 8.69E-05 |
| TRINITY_DN4660_c0_g1 | GO:0005829,root meristem specification | 8.71E-05 |
| TRINITY_DN4683_c0_g1 | GO:0005524,root system development | 8.87E-05 |
| TRINITY_DN15_c2_g1 | GO:0031410,root development | 9.23E-05 |
| TRINITY_DN4681_c0_g1 | GO:0009986,lateral root formation | 9.43E-05 |
| TRINITY_DN4653_c0_g1 | GO:0005829,root hair elongation | 9.45E-05 |
| TRINITY_DN4611_c0_g1 | GO:0005829,root epidermal cell differentiation | 9.45E-05 |
| TRINITY_DN4637_c0_g1 | GO:0016021,response to water deprivation`,root development | 9.47E-05 |
| TRINITY_DN4631_c0_g1 | GO:0009507,response to salt stress,root development | 9.7E-05 |
| TRINITY_DN4682_c1_g1 | GO:0016021,regulation of root meristem growth | 9.7E-05 |
| TRINITY_DN4604_c0_g2 | GO:0005856,lateral root formation | 9.87E-05 |
| TRINITY_DN4600_c0_g1 | GO:0016021,response to water deprivation`root development | 0.0001 |
| TRINITY_DN62_c0_g2 | GO:0048046,root development` | 0.000103 |
| TRINITY_DN79_c0_g1 | GO:0005783,root development | 0.000104 |
| TRINITY_DN94_c0_g1 | GO:0005886,root development | 0.000105 |
| TRINITY_DN60_c0_g1 | GO:0005634,root morphogenesis | 0.000106 |
| TRINITY_DN99_c0_g2 | GO:0005739,root development | 0.000107 |
| TRINITY_DN4602_c0_g2 | GO:0016021,response to water deprivation`root development | 0.000108 |
| TRINITY_DN92_c1_g1 | GO:0005739,root development | 0.000108 |
| TRINITY_DN54_c0_g1 | GO:0016021,root development | 0.00011 |
| TRINITY_DN58_c0_g1 | GO:0009507,lateral root development, negative regulation of response to water deprivation | 0.000113 |
| TRINITY_DN91_c0_g2 | GO:0009504,root hair initiation | 0.000114 |
| TRINITY_DN52_c2_g1 | GO:0016021,response to water deprivation,root development | 0.000114 |
| TRINITY_DN4619_c0_g1 | GO:0005737,root development` | 0.000115 |
| TRINITY_DN83_c0_g1 | GO:0005834,regulation of root development,response to ethylene | 0.000124 |
| TRINITY_DN88_c0_g1 | GO:0015629,root hair elongation | 0.000125 |
| TRINITY_DN53_c0_g1 | GO:0005829,root hair initiation | 0.000125 |
| TRINITY_DN42_c0_g2 | GO:0005634,lateral root development`response to sucrose | 0.000126 |
| TRINITY_DN5_c0_g1 | GO:0016324,root hair initiation | 0.00016 |
| TRINITY_DN4656_c0_g1 | GO:0048046,negative regulation of cell growth,root development | 0.001417 |

**Table S5. Significantly enriched KEGG pathways in the DEGs of IND 04-1335 under drought stress (FDR<0.01)**

| **KEGG Pathway** | **ID** | **Input number** | **P-Value** | **FDR** |
| --- | --- | --- | --- | --- |
| Metabolic pathways | Ko01100 | 1173 | 3.96E-90 | 4.9E-88 |
| Biosynthesis of secondary metabolites | Ko01110 | 642 | 7.79E-53 | 4.83E-51 |
| Ribosome | Ko03010 | 266 | 1.72E-47 | 7.1E-46 |
| Phenylpropanoid biosynthesis | Ko00940 | 187 | 9.71E-31 | 3.01E-29 |
| Carbon metabolism | Ko01200 | 169 | 5.23E-23 | 1.3E-21 |
| Glycolysis / Gluconeogenesis | Ko00010 | 109 | 3.75E-19 | 7.75E-18 |
| Cysteine and methionine metabolism | Ko00270 | 90 | 4.82E-18 | 8.54E-17 |
| Carbon fixation in photosynthetic organisms | Ko00710 | 73 | 1.03E-16 | 1.59E-15 |
| Biosynthesis of amino acids | Ko01230 | 123 | 4.52E-12 | 6.23E-11 |
| Galactose metabolism | Ko00052 | 57 | 5.92E-12 | 7.34E-11 |
| Pyrimidine metabolism | Ko00240 | 50 | 2.69E-10 | 3.04E-09 |
| Pyruvate metabolism | Ko00620 | 55 | 5.43E-08 | 5.61E-07 |
| Pentose phosphate pathway | Ko00030 | 42 | 8.08E-08 | 7.71E-07 |
| Amino sugar and nucleotide sugar metabolism | Ko00520 | 77 | 1.58E-07 | 1.37E-06 |
| Nitrogen metabolism | Ko00910 | 31 | 1.66E-07 | 1.37E-06 |
| beta-Alanine metabolism | Ko00410 | 38 | 1.89E-07 | 1.47E-06 |
| Valine, leucine and isoleucine degradation | Ko00280 | 37 | 2.13E-07 | 1.55E-06 |
| Starch and sucrose metabolism | Ko00500 | 81 | 4.36E-07 | 3.01E-06 |
| Fructose and mannose metabolism | Ko00051 | 43 | 6.54E-07 | 4.27E-06 |
| MAPK signaling pathway - plant | Ko04016 | 74 | 7.2E-07 | 4.47E-06 |
| Propanoate metabolism | Ko00640 | 30 | 9.57E-07 | 5.65E-06 |
| Alanine, aspartate and glutamate metabolism | Ko00250 | 35 | 3E-06 | 1.69E-05 |
| Citrate cycle (TCA cycle) | Ko00020 | 35 | 4.11E-06 | 2.21E-05 |
| Arginine and proline metabolism | Ko00330 | 37 | 5.45E-06 | 2.81E-05 |
| DNA replication | Ko03030 | 40 | 1.62E-05 | 8.04E-05 |
| Protein processing in endoplasmic reticulum | Ko04141 | 82 | 0.000118 | 0.000564 |
| Fatty acid degradation | Ko00071 | 31 | 0.00013 | 0.000598 |
| Glutathione metabolism | Ko00480 | 55 | 0.000148 | 0.000653 |
| Glyoxylate and dicarboxylate metabolism | Ko00630 | 35 | 0.000162 | 0.000694 |
| Plant-pathogen interaction | Ko04626 | 69 | 0.000212 | 0.000876 |
| Homologous recombination | Ko03440 | 38 | 0.000325 | 0.001299 |
| Mismatch repair | Ko03430 | 30 | 0.000405 | 0.001569 |
| Taurine and hypotaurine metabolism | Ko00430 | 14 | 0.000829 | 0.003117 |
| Inositol phosphate metabolism | Ko00562 | 31 | 0.000971 | 0.003542 |
| Phagosome | Ko04145 | 35 | 0.001183 | 0.004189 |
| Ascorbate and aldarate metabolism | Ko00053 | 25 | 0.001278 | 0.004403 |
| Phenylalanine metabolism | Ko00360 | 23 | 0.001472 | 0.004934 |
| Circadian rhythm - plant | Ko04712 | 27 | 0.001674 | 0.005463 |
| Glycerolipid metabolism | Ko00561 | 41 | 0.001739 | 0.00553 |
| Monobactam biosynthesis | Ko00261 | 10 | 0.002844 | 0.008817 |
| Phosphatidylinositol signaling system | Ko04070 | 31 | 0.002931 | 0.008864 |

**Table S6. Significantly enriched KEGG pathways in the DEGs of CO 86032 under drought stress (FDR<0.01)**

| **KEGG pathway** | **ID** | **Input number** | **P-Value** | **FDR** |
| --- | --- | --- | --- | --- |
| Metabolic pathways | Ko01100 | 1050 | 8.33767E-64 | 1.03387E-61 |
| Protein processing in endoplasmic reticulum | Ko04141 | 146 | 1.90562E-22 | 1.18149E-20 |
| Biosynthesis of amino acids | Ko01230 | 148 | 6.05635E-20 | 2.50329E-18 |
| Biosynthesis of secondary metabolites | Ko01110 | 475 | 2.93184E-18 | 9.08869E-17 |
| Spliceosome | Ko03040 | 123 | 8.43561E-16 | 2.09203E-14 |
| Carbon metabolism | Ko01200 | 143 | 1.03279E-15 | 2.13444E-14 |
| Citrate cycle (TCA cycle) | Ko00020 | 52 | 6.7955E-13 | 1.08177E-11 |
| Phosphatidylinositol signaling system | Ko04070 | 60 | 6.97916E-13 | 1.08177E-11 |
| Alanine, aspartate and glutamate metabolism | Ko00250 | 51 | 1.12696E-12 | 1.52367E-11 |
| RNA transport | Ko03013 | 101 | 1.22877E-12 | 1.52367E-11 |
| 2-Oxocarboxylic acid metabolism | Ko01210 | 49 | 7.42803E-11 | 8.37342E-10 |
| Endocytosis | Ko04144 | 90 | 1.74175E-10 | 1.79981E-09 |
| Inositol phosphate metabolism | Ko00562 | 51 | 2.07647E-10 | 1.98064E-09 |
| Pyrimidine metabolism | Ko00240 | 49 | 3.78808E-10 | 3.35516E-09 |
| Proteasome | Ko03050 | 47 | 6.89851E-10 | 5.70277E-09 |
| mRNA surveillance pathway | Ko03015 | 67 | 1.12272E-08 | 8.70105E-08 |
| Ribosome | Ko03010 | 135 | 2.54403E-08 | 1.85565E-07 |
| Aminoacyl-tRNA biosynthesis | Ko00970 | 43 | 3.19641E-08 | 2.20197E-07 |
| Phagosome | Ko04145 | 48 | 1.84854E-07 | 1.20642E-06 |
| Valine, leucine and isoleucine degradation | Ko00280 | 36 | 3.35624E-07 | 2.08087E-06 |
| DNA replication | Ko03030 | 44 | 6.46141E-07 | 3.81531E-06 |
| N-Glycan biosynthesis | Ko00510 | 33 | 1.02129E-06 | 5.75634E-06 |
| Glycolysis / Gluconeogenesis | Ko00010 | 68 | 1.21303E-06 | 6.5398E-06 |
| Autophagy - other | Ko04136 | 31 | 1.32356E-06 | 6.83837E-06 |
| Mismatch repair | Ko03430 | 37 | 2.0843E-06 | 1.01835E-05 |
| Pyruvate metabolism | Ko00620 | 49 | 2.13525E-06 | 1.01835E-05 |
| Arginine biosynthesis | Ko00220 | 26 | 3.44613E-06 | 1.58267E-05 |
| Butanoate metabolism | Ko00650 | 23 | 4.44923E-06 | 1.97037E-05 |
| Ribosome biogenesis in eukaryotes | Ko03008 | 50 | 5.60936E-06 | 2.39849E-05 |
| Cysteine and methionine metabolism | Ko00270 | 53 | 6.08534E-06 | 2.51528E-05 |
| RNA degradation | Ko03018 | 57 | 6.46732E-06 | 2.58693E-05 |
| Plant-pathogen interaction | Ko04626 | 75 | 7.80358E-06 | 3.02262E-05 |
| Glycine, serine and threonine metabolism | Ko00260 | 39 | 8.04408E-06 | 3.02262E-05 |
| Sulfur metabolism | Ko00920 | 26 | 1.09261E-05 | 3.9848E-05 |
| Ubiquitin mediated proteolysis | Ko04120 | 63 | 1.12566E-05 | 3.98805E-05 |
| Tyrosine metabolism | Ko00350 | 33 | 1.33758E-05 | 4.60724E-05 |
| beta-Alanine metabolism | Ko00410 | 32 | 1.587E-05 | 5.31858E-05 |
| Nucleotide excision repair | Ko03420 | 41 | 3.05069E-05 | 9.9549E-05 |
| Glyoxylate and dicarboxylate metabolism | Ko00630 | 37 | 3.19722E-05 | 0.000101655 |
| Purine metabolism | Ko00230 | 48 | 3.53617E-05 | 0.000109621 |
| MAPK signaling pathway - plant | Ko04016 | 64 | 7.77255E-05 | 0.000235072 |
| Sphingolipid metabolism | Ko00600 | 26 | 0.000107136 | 0.000316307 |
| Arginine and proline metabolism | Ko00330 | 32 | 0.000132368 | 0.000381714 |
| Basal transcription factors | Ko03022 | 27 | 0.000156937 | 0.000442276 |
| Oxidative phosphorylation | Ko00190 | 58 | 0.000263282 | 0.000725489 |
| Phenylalanine metabolism | Ko00360 | 25 | 0.000296247 | 0.000798578 |
| Phenylpropanoid biosynthesis | Ko00940 | 89 | 0.000326028 | 0.000860159 |
| Homologous recombination | Ko03440 | 37 | 0.000422641 | 0.001091823 |
| Starch and sucrose metabolism | Ko00500 | 65 | 0.000575806 | 0.001457141 |
| Isoquinoline alkaloid biosynthesis | Ko00950 | 19 | 0.0006564 | 0.001627871 |
| Glycerolipid metabolism | Ko00561 | 42 | 0.000784658 | 0.001907795 |
| Propanoate metabolism | Ko00640 | 21 | 0.001061819 | 0.00253203 |
| Nitrogen metabolism | Ko00910 | 20 | 0.001283116 | 0.003002008 |
| Fatty acid biosynthesis | Ko00061 | 27 | 0.001328831 | 0.003022014 |
| Galactose metabolism | Ko00052 | 31 | 0.001340409 | 0.003022014 |
| Cyanoamino acid metabolism | Ko00460 | 31 | 0.002301783 | 0.005096806 |
| Tropane, piperidine and pyridine alkaloid biosynthesis | Ko00960 | 16 | 0.002728113 | 0.005934843 |
| Amino sugar and nucleotide sugar metabolism | Ko00520 | 56 | 0.002858365 | 0.006110987 |
| Histidine metabolism | Ko00340 | 13 | 0.004763756 | 0.010011962 |
